# SOX9 regulated matrix proteins are increased in patients serum and correlate with severity of liver fibrosis

**DOI:** 10.1038/s41598-018-36037-4

**Published:** 2018-12-17

**Authors:** Varinder S. Athwal, James Pritchett, Katherine Martin, Jessica Llewellyn, Jennifer Scott, Emma Harvey, Abed M. Zaitoun, Aoibheann F. Mullan, Leo A. H. Zeef, Scott L. Friedman, William L. Irving, Neil A. Hanley, Indra N. Guha, Karen Piper Hanley

**Affiliations:** 1 Wellcome Centre for Cell-Matrix Research, Faculty of Biology, Medicine & Health, Manchester Academic Health Science Centre, University of Manchester, Oxford Road, Manchester, M13 9PT UK; 2 Division of Diabetes, Endocrinology and Gastroenterology, Faculty of Biology, Medicine & Health, University of Manchester, Manchester Academic Health Science Centre, Oxford Road, Manchester, UK; 3 Research & Innovation Division, Central Manchester University Hospitals NHS Foundation Trust, Oxford Road, Manchester, M13 9PT UK; 4 School of Healthcare Science, Manchester Metropolitan University, Manchester, M1 5GD UK; 5 Department of Cellular Pathology, NIHR Biomedical Research Centre, Nottingham University Hospitals NHS Trust and University of Nottingham, Nottingham, UK; 6 Bioinformatics Core Facility, Faculty of Life Sciences, University of Manchester, Manchester, UK; 7 Division of Liver Diseases, Icahn School of Medicine at Mount Sinai, New York, NY10029 USA; 8 NIHR Biomedical Research Centre, Nottingham University Hospitals NHS Trust and University of Nottingham, Nottingham, UK; 9 School of Life Sciences, NIHR Biomedical Research Centre, Nottingham University Hospitals NHS Trust and University of Nottingham, Nottingham, UK

**Keywords:** Physiology, Gastroenterology

## Abstract

Extracellular matrix (ECM) deposition and resultant scar play a major role in the pathogenesis and progression of liver fibrosis. Identifying core regulators of ECM deposition may lead to urgently needed diagnostic and therapetic strategies for the disease. The transcription factor Sex determining region Y box 9 (SOX9) is actively involved in scar formation and its prevalence in patients with liver fibrosis predicts progression. In this study, transcriptomic approaches of *Sox9*-abrogated myofibroblasts identified >30% of genes regulated by SOX9 relate to the ECM. Further scrutiny of these data identified a panel of highly expressed ECM proteins, including Osteopontin (OPN), Osteoactivin (GPNMB), Fibronectin (FN1), Osteonectin (SPARC) and Vimentin (VIM) as SOX9 targets amenable to assay in patient serum. *In vivo* all SOX-regulated targets were increased in human disease and mouse models of fibrosis and decreased following *Sox9*-loss in mice with parenchymal and biliary fibrosis. In patient serum samples, SOX9-regulated ECM proteins were altered in response to fibrosis severity, whereas comparison with established clinical biomarkers demonstrated superiority for OPN and VIM at detecting early stages of fibrosis. These data support SOX9 in the mechanisms underlying fibrosis and highlight SOX9 and its downstream targets as new measures to stratify patients with liver fibrosis.

## Introduction

Liver fibrosis is increasing and a major cause of morbidity and mortality^1–3^. It is a feature of most chronic liver diseases and is characterized by progressive deposition of extracellular matrix (ECM) proteins resulting in pathological scaring and tissue dysfunction^4^. Although potentially reversible during early stages, a significant number of patients progress to advanced fibrosis and end-stage cirrhosis, increasing the risk of hepatocellular carcinoma (HCC)^5–7^. Identifying the extent of fibrosis and risk of progression would provide a valuable clinical tool.

Liver biopsy remains an important measure to assess fibrosis. However, several biological tests already in clinical use have taken advantage of secreted factors associated with the pathogenesis of liver fibrosis including ECM remodeling (Tissue inhibitor of metalloproteinases-1; TIMP1) and deposition (Procollagen III propeptide; PIIIP)^8–11^. Consequently core regulators of ECM deposition could be exploited to identify useful targets for urgently needed diagnostic and therapetic strategies for the disease. We have previously identified the transcription factor, Sex determining region Y box 9 (SOX9), as a key factor regulating multiple components of the fibrotic ECM in liver disease whereas its prevalence in patient biopsy samples predicts progression towards cirrhosis^12–16^.

These studies stem from SOX9’s critical role in bone development and its requirement in chondrogenesis, whereby the initial cartilaginous skeletal elements are formed and serve as a template for endochondral bone formation^14,17,18^. Here SOX9 transcriptionally activates many cartilage-specific ECM genes such as Collagens type-2, 9, 11 and 27, Aggrecan, Matrillin-1 and Cartilage Oligomeric Protein^14,17–19^. Importantly, SOX9 is silenced in terminally differentiated chondrocytes prior to ossification^17^. This context-specific expression of SOX9 is mediated by several signaling pathway, many of which become dysregulated in fibrotic disease^4,14^. In light of this and in response to profibrotic signaling factors, SOX9 becomes expressed by the fibrogenic cell-type, the activated hepatic stellate cell (HSC), a myofibroblast that contains α-smooth muscle actin (αSMA) and regulates the production of ECM components, type 1 Collagen (COL1) and OPN^13,15,16^, both implicated in disease progression^20,21^, and inhibits the collagenase Matrix metalloproteinase-13 (MMP13)^15^. In the context of fibrosis and increased organ stiffness, studies in development, liver fibrosis and regeneration all support mechanisms involving the mechanosenstive factor Yes associated protein-1 (YAP-1) as important in the regulation of SOX9^22–24^. *In vivo*, mice lacking SOX9 have significantly reduced scarring, improved liver function and less inflammation in models of fibrosis^12^. Data from multiple transgenic models to inactivate SOX9 support hepatic myofibroblasts as the causative cell-type mediating fibrosis^12^. In human patients with chronic liver disease, the profile and localization of SOX9 was identical to rodent suggesting the mechanisms underlying SOX9 function in fibrosis are likely to be the same^12^. Moreover, the extent of SOX9 in biopsies from patients with chronic liver disease correlated with fibrosis severity and accurately predicted disease progression towards cirrhosis^12^.

As an extension to these data placing SOX9 in the mechanism underlying fibrosis, we hypothesized that defining downstream SOX9 targets in liver myofibroblasts would identify ECM components amenable to assay in serum from patients with chronic liver disease. Through transcriptomic and experimental analysis of *Sox9*-abrogated and wild-type liver myofibroblasts, gene ontology and functional analysis identified >30% of genes regulated by SOX9 relate to the ECM. Further scrutiny of these data identified a panel of highly expressed ECM proteins, verified *in vitro* and *in vivo* as SOX9 targets that correlated with severity of fibrosis in patient serum samples.

## Results

### SOX9 regulates multiple ECM proteins in HSCs

**Figure 1:**
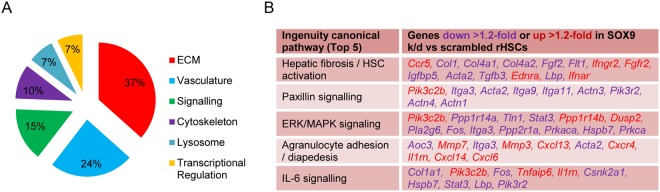
SOX9 regulates multiple ECM proteins in HSCs. (**A**) Function annotation for gene ontology of all *Sox9*-regulated genes (±1.2 fold, p < 0.05) represented as proportion of individual categories outlined and listed in Supplementary Figure 2. (**B**) Top 5 canonical pathways represented and *Sox9*-regulated genes listed. Down-regulated genes (purple) and up-regulated (red) are highlighted following Sox9-loss.

Transcriptome analysis of *Sox9* depleted activated rat HSCs (ArHSCs) compared to control cells resulted in a total of 540 differentially regulated genes (± ≥ 1.2 fold, p < 0.05; Supplementary Figure 1A-B, Supplementary Information File 1 and microarray dataset E-MTAB-7298). Verification of the top 15 SOX9-regulated genes indicated 87% of positive targets were in line with our microarray data (Supplementary Fig. 1C,D). Gene ontology and functional annotation analysis revealed 37% of genes regulated by SOX9 related to the ECM (Fig. 1 and Supplementary Figs 2 and 3). Combined with our previous data and its ability to transcriptionally activate a repertoire of ECM genes during chondrogenesis^13,15,16,25^, these data support SOX9’s central role in fibrotic mechanisms resulting in scar formation and subsequent tissue destruction^12^.

Further scrutiny of this dataset discovered a cohort of highly expressed genes, with transcript levels above the threshold levels for the microarray platform to perform an assay. Significantly, this group contained known SOX9 regulated genes, *Opn* (or *Spp1*) and *Col1*^13,16^. As a result, we were interested to determine whether other matrix proteins were similarly regulated by SOX9 in these data. From 169 highly expressed genes (with ≥3800 arbitrary units of expression), 41% of transcripts encoded ribosomal proteins; further highlighting the abundant levels of the remaining genes in activated HSCs (Supplementary Table 3). Following ribosomal gene removal, from the remaining 100 transcripts (Supplementary Table 3) we identified a subgroup of 24 genes encoding functional proteins emphasizing the phenotype of activated HSCs (Table 1). This list contained genes relevant to pro-inflammatory ligands and contractile cytoskeletal factors (Table 1). However, 10 genes encoded secreted matrix proteins potentially amenable to assay in patient serum samples (asterisks; Table 1).

**Table 1:** Subgroup of highly expressed HSC transcripts encoding secreted proteins.

Gene Symbol	Gene Title
Vim*	vimentin
Gpnmb*	glycoprotein transmembrane nmb (osteoactivin)
Spp1*	secreted phosphoprotein 1 (osteopontin)
Ctgf*	connective tissue growth factor
*Ccl2*	chemokine (C-C motif) ligand 2
*Pf4*	platelet factor 4/chemokine (C-X-C motif) ligand 4
*Txn1*	thioredoxin 1
*Ccl7*	chemokine (C-C motif) ligand 7
*Tpm2*	tropomyosin 2
*Ctsl1*	cathepsin L1
*Ctsb*	cathepsin B
Mgp*	matrix Gla protein
Tagin*	Transgelin
*Cxcl3*	chemokine (C-X-C motif) ligand 3
Sparc*	secreted protein, acidic, cysteine-rich (osteonectin)
*Cd63*	Cd63 molecule
*Cox2*	cyclo-oxygenase 2
*Cxcl3*	chemokine (C-X-C motif) ligand 3
*Eno1*	enolase 1, (alpha Enolase)
Timp1*	Tissue metallopeptidase inhibitor 1
*Cryab*	crystallin, alpha B
Col1a2*	collagen, type I, alpha 2
*Tpm4*	tropomyosin 4
Fn1*	fibronectin 1

**Figure 2:**
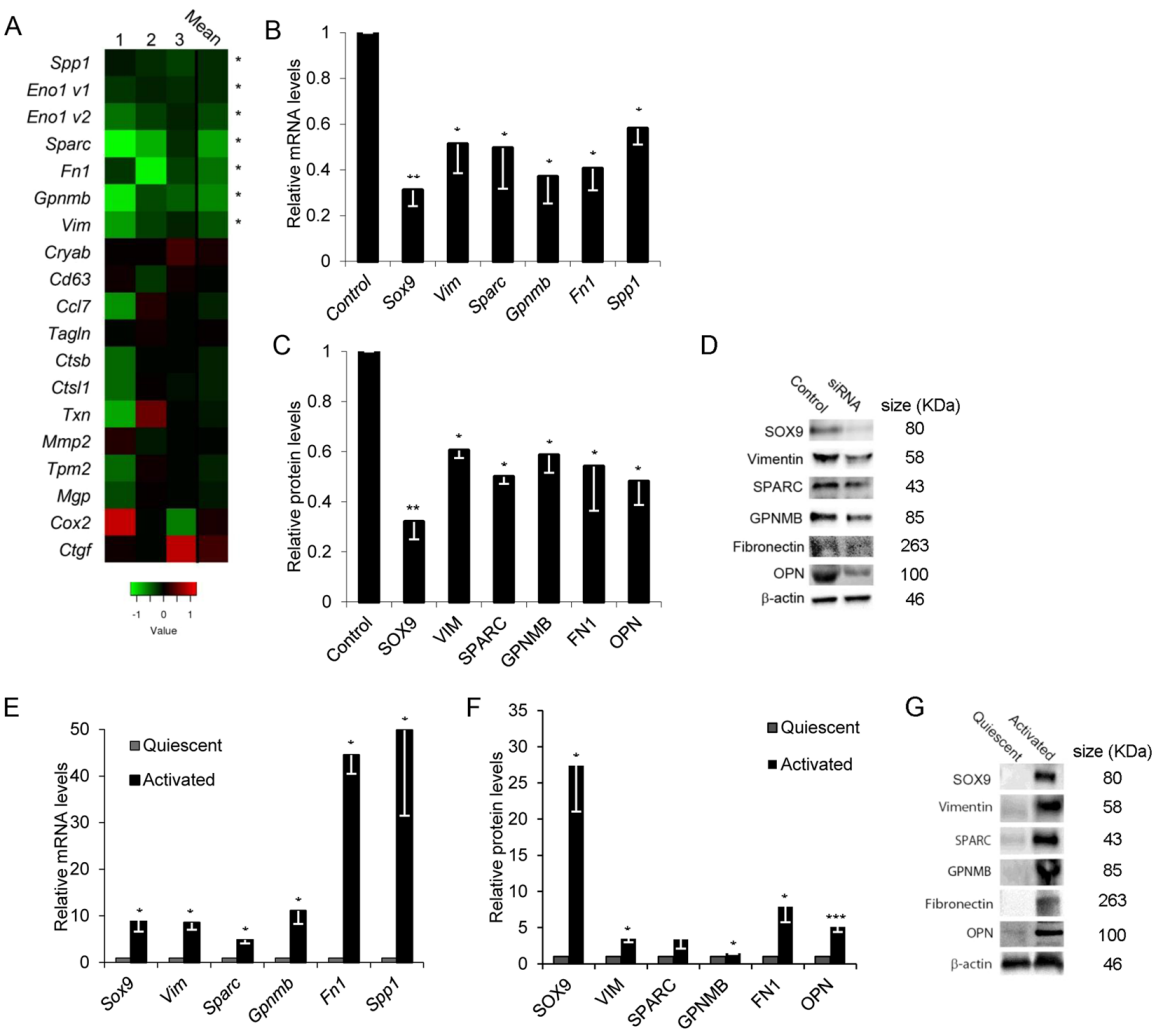
Identification of a panel of *Sox9*-regulated genes in HSCs. (**A**) Comparative levels and representation of highly expressed factors after knockdown of *Sox9* in activated rat HSCs (*in A signifies statistically significant change). Relative mRNA levels by qRT-PCR analysis and protein levels quantified by immunoblotting in rHSCs following *Sox9* abrogation using siRNA (**B**) and (**C**), representative immunoblot shown in (**D**) or quiescent and activated rHSCs (E) and (F, representative immunoblot shown in (**G**). All experiments are n ≥3. Data are shown as means ± s.e.m. *p < 0.05, **p < 0.01, ***p < 0.001.

**Figure 3:**
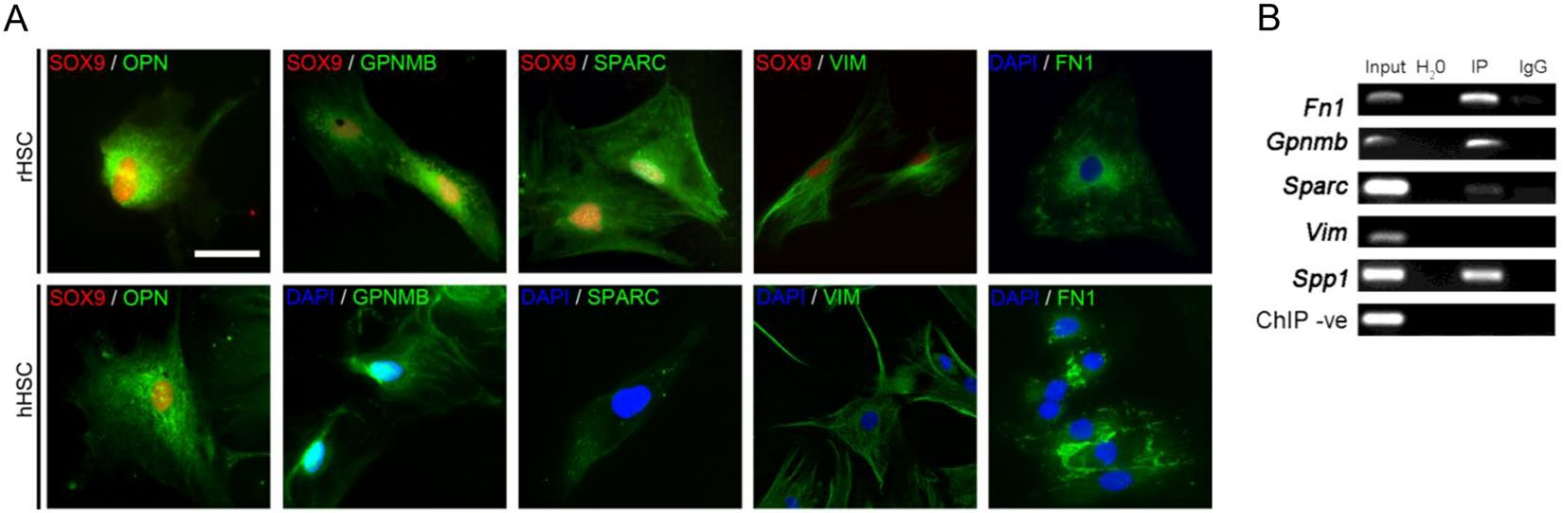
SOX9 is localized and directly binds to its ECM targets in HSCs. (**A**) Immunofluorescence in activated HSCs from rat (rHSCs; top panel) and human (hHSCs; bottom panel). Nuclear SOX9 shown in red with OPN, GPNMB, SPARC, VIM and FN1 shown in green. Co-localisation is shown where antibody compatibility allows. DAPI nuclear stain (blue). Scale bar 10 μm. (**B**) ChIP assay for SOX9 binding element in primary rat HSCs with negative controls (IgG and ChIP negative primers) and positive control (Input diluted 1:10). n = 3. SOX9 enrichment (IP) is shown for *Fn1*, *Gpnmb*, *Sparc* and *Spp1* (*Opn*).

To evaluate if the identified factors were regulated by SOX9, we abrogated SOX9 expression in activated HSCs using siRNA (Fig. 2A). A reduction in mRNA and protein levels of 5 SOX9 targets, namely *Opn*, *Gpnmb*, *Fn1*, *Sparc*, and *Vim* was uncovered (Fig. 2B–D). Compared to quiescent HSC controls, transcript and protein levels were increased in activated HSCs (Fig. 2E–G). *Eno1* (Enolase) expression was significantly reduced by *Sox9* knockdown (Fig. 2A), but did not show any change in activated HSCs compared to quiescent controls (data not shown) and hence not pursued. Immunocytochemistry showed presence of all five factors in activated rat and human HSCs (Fig. 3A).

Similar to our previous work on *Opn*^16^*, in-silico* analysis identified conserved SOX9 binding motifs in *Gpnmb*, *Fn1*, *Sparc* and *Vim* within regions <3Kb upstream of the transcriptional start site (Supplementary Figure 4). Consistent with this^16^, *Opn, Gpnmb, Fn1* and *Sparc* enrichment was demonstrated in activated rHSCs following ChIP with a SOX9 antibody (Fig. 3B), whereas *Vim* did not appear to be directly regulated by SOX9 through this binding site.

### SOX9-targets detected in fibrotic areas in mouse and human and reduced following Sox9-loss

**Figure 4:**
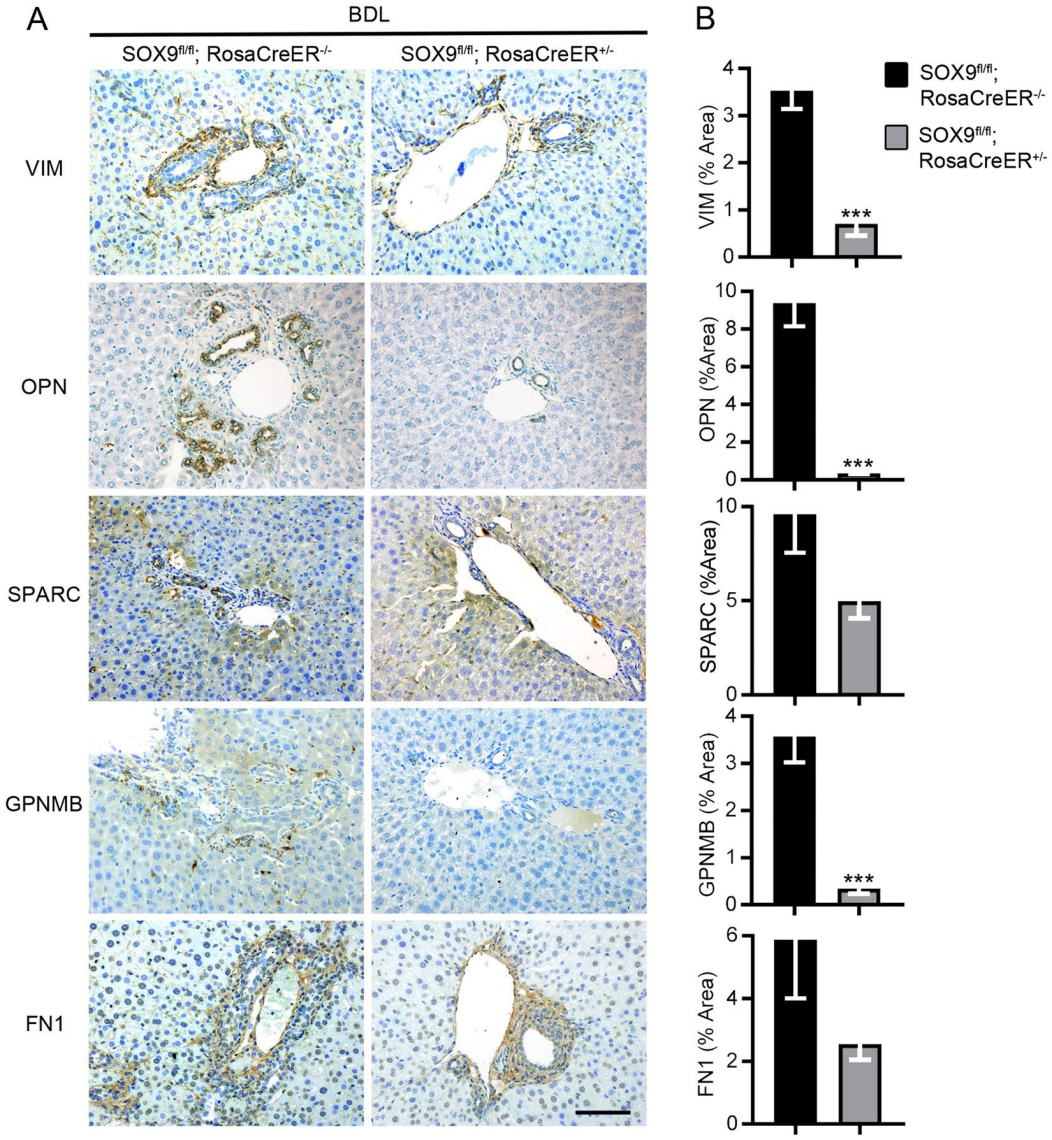
Localization and quantification of SOX9-regulated proteins in fibrotic liver following *Sox9*-loss in mice with BDL-induced fibrosis. (**A**) Immunohistochemistry for VIM, OPN, SPARC, GPNMB and FN1 (brown) counterstained with toludine blue. Images shown for control (Sox9fl/fl; RosaCreER−/−) and *Sox9*-null (Sox9fl/fl; RosaCreER+/−) livers following CCl_4_ induced fibrosis. Scale bar 100 μm. (**B**) Quantification of surface area covered by individual protein staining in control (Sox9fl/fl; RosaCreER−/−) and *Sox9*-null (Sox9fl/fl; RosaCreER+/−) livers in (**A**). All experiments are n = 5. Data are shown as means ± s.e.m. ***p < 0.001.

**Figure 5:**
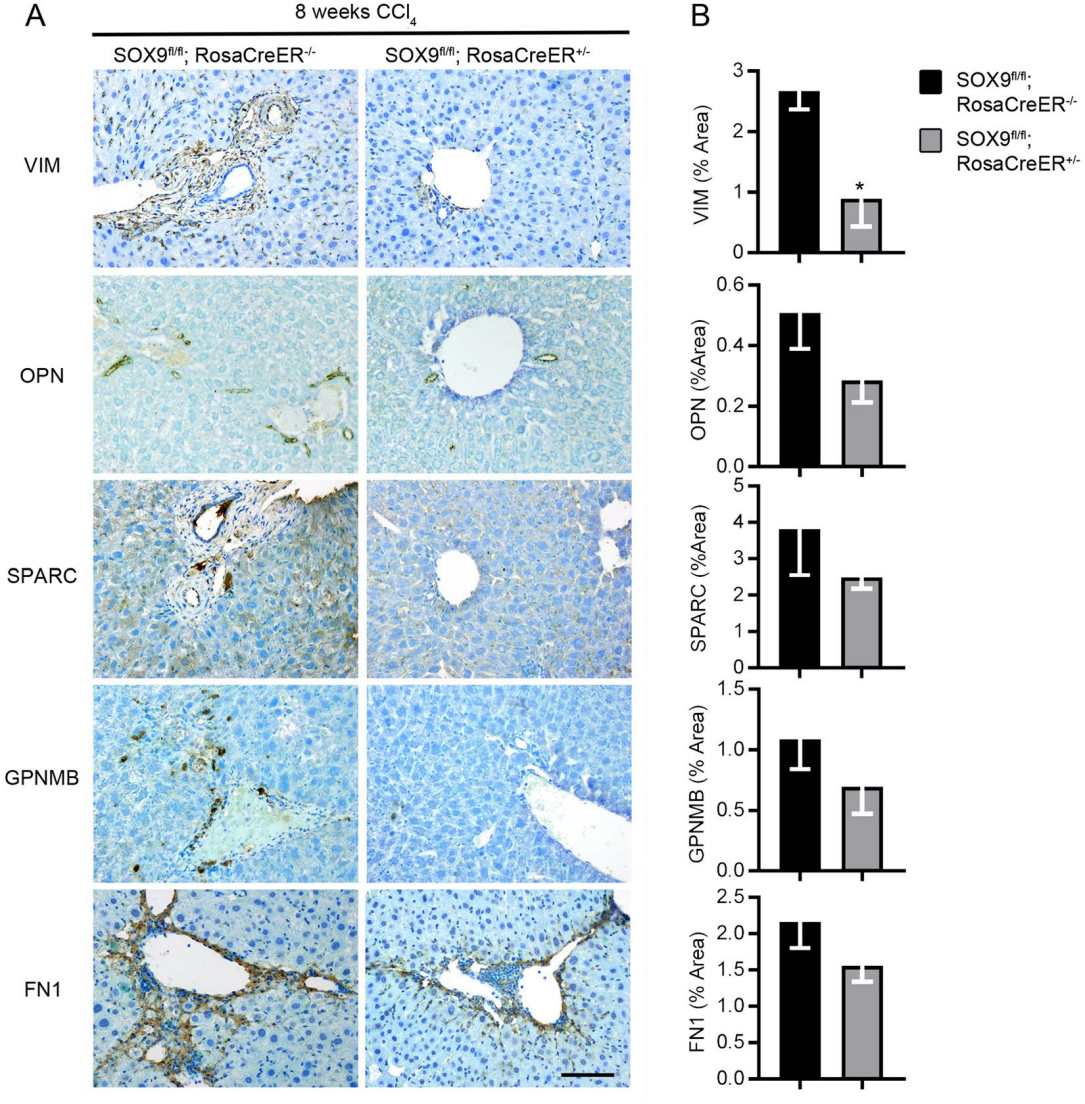
Localization and quantification of SOX9-regulated proteins in fibrotic liver following *Sox9*-loss in mice with CCl_4_ induced fibrosis. (**A**) Immunohistochemistry for VIM, OPN, SPARC, GPNMB and FN1 (brown) counterstained with toludine blue. Images shown for control (Sox9fl/fl; RosaCreER−/−) and *Sox9*-null (Sox9fl/fl; RosaCreER+/−) livers following BDL induced fibrosis. Scale bar 100 μm. (**B**) Quantification of surface area covered by individual protein staining in control (Sox9fl/fl; RosaCreER−/−) and *Sox9*-null (Sox9fl/fl; RosaCreER+/−) livers in (**A**). All experiments are n = 5. Data are shown as means ± s.e.m. *p < 0.05.

**Figure 6:**
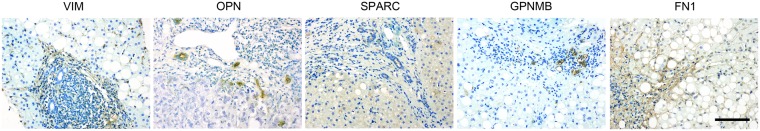
Localization of SOX9-regulated proteins in fibrotic liver from human. Immunohistochemistry for VIM, OPN, SPARC, GPNMB and FN1 (brown) counterstained with toludine blue. Images shown for advanced fibrotic/cirrhotic human liver secondary to CHC infection. Scale bar 100 μm.

We have previously described improved fibrosis in *Sox9*-null mice^12^. In line with this, expression of all targets were increased following fibrosis induction by CCl_4_ and BDL in control mice (Sox9fl/fl; RosaCreER−/−), whereas all were reduced following *Sox9*-loss (Sox9fl/fl; RosaCreER+/−) (Figs 4 and 5 and Supplementary Figs 5 and 6). Similar to mouse, liver biopsy samples from patients with advanced fibrosis secondary to chronic hepatitis C (CHC) showed high levels of all identified SOX9 targets in advanced fibrosis/cirrhosis (IS6; Fig. 6 and Supplementary Fig. 7). In line with our previous work and rodent models of fibrosis (Supplementary Figs 8 and 9), the patient samples expressed increased levels of SOX9 and α-SMA (Supplementary Fig. 7)^12^.

### Analysis of the SOX9 ECM panel as markers of liver fibrosis

These data suggested the SOX9 downstream targets, OPN, VIM, SPARC, GPNMB and FN1, were secreted from activated HSCs and increased in fibrotic disease. To ascertain clinical significance, we investigated whether SOX9 regulated proteins could be used to assess fibrosis in serum samples from a well phenotyped, experimental cohort of patients with CHC (and non-infected controls). This cohort consisted of 50 patients with parallel serum and liver biopsy samples. Fibrosis was assessed by biopsies and staged using the Metavir system. Breakdown by fibrosis stage was: controls (n = 11), F0 (n = 6), F1 (n = 12), F2 (n = 9), F3 (n = 1) and F4/cirrhosis (n = 11). With only a single sample from a patient with F3 fibrosis, this was amalgamated into F4 as representative analysis of clinically advanced fibrosis.

**Figure 7:**
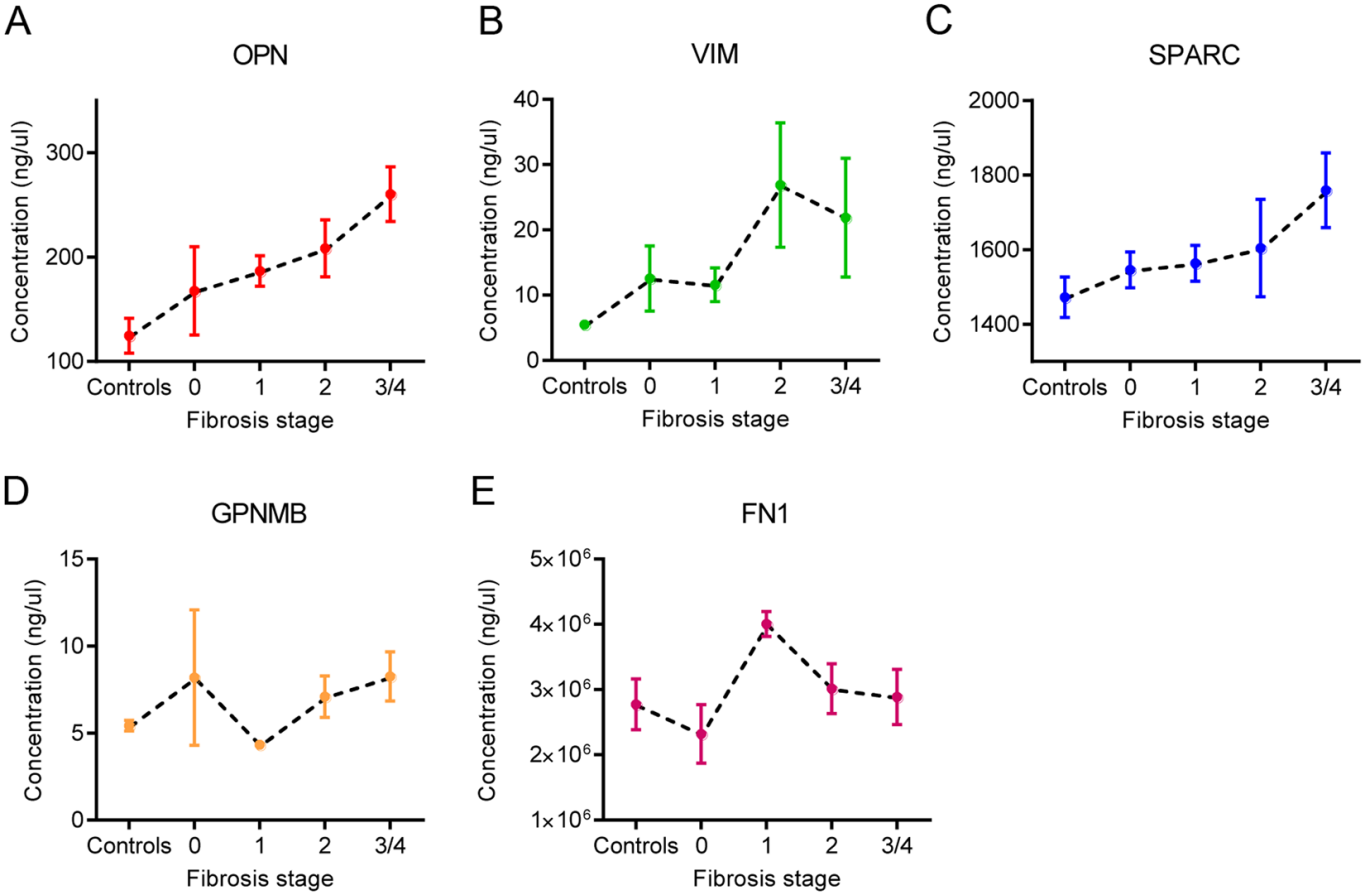
SOX9-regulated proteins are present and increased in serum from patients with liver fibrosis. (**A**–**E**) Serum concentration of SOX9-regulated targets quantified by ELISA and grouped by stage of fibrosis (Metavir). Data are shown as means ± s.e.m.

Concentration of individual proteins was assayed in the serum samples using ELISA based immunoassay. Assay consistency and accuracy was assessed using the average coefficient of variation (CoV). For each protein assayed, CoV was: OPN – 6.66%, VIM – 4.74%, SPARC 4.60%, GPNMB 6.01% and FN1–8.20%. A CoV below 10% is laboratory standard for accuracy and all ELISAs fulfilled this criterion. Serum concentrations from three of the five tested proteins had promising performance to predict fibrosis stage (Fig. 7). Concentrations of OPN, VIM and SPARC increased in a step-wise manner, although VIM concentrations did not alter significantly between latter fibrosis stages F2–4 (Fig. 7). Similar results were observed in a second HCV cohort, staged by the 7-point Ishak (IS) fibrosis stage^26^, IS0 to IS6 (the latter representing the most severe fibrosis/cirrhosis) comparing no fibrosis (F0; n = 52), mild (IS1–2; n = 60) versus severe (IS5–6; n = 19) fibrosis (Supplementary Figure 10).

In the experimental cohort, AUROC distinguished performance of individual experimental biomarkers (Table 2). These data identified OPN, VIM, GPNMB and SPARC with the best diagnostic performance in discriminating between non-cirrhotic (F0-F3) and cirrhosis (F4) with AUROCs of OPN 0.78 [CI 0.62, 0.94], VIM 0.71 [CI 0.54, 0.87], GPNMB 0.73 [CI 0.56, 0.91] and SPARC 0.70 [CI 0.501, 0.90] (Table 2). In comparison to OPN and VIM that had consistent performance across different stages of fibrosis (though both show superiority at earlier fibrosis stages), GPNMB had diagnostic ability in latter stages of fibrosis (AUROC of >0.7). SPARC AUROC values were inferior to both OPN and VIM throughout the range of fibrosis. FN1 performed poorly, values peaked at F1 fibrosis and then returned to near baseline levels as fibrosis progressed (Fig. 5). This would suggest an unfavorable potential as a biomarker. However, the discriminatory ability of FN1 level to predict F1 fibrosis is high (AUROC 0.81 [CI 0.67–0.94]), and could potentially make FN1 serum concentration useful in a biomarker panel.

**Table 2:** AUROC analysis.

	AUROC: Experimental cohort (n = 50)									
	Experimental biomarkers					Previously validated biomarkers & biomarker panels				
Fibrosis Stage	OPN	VIM	SPARC	GPNMB	FN1	APRI	HA	P3NP	TIMP1	ELF
C vs 0–4	0.797	0.876	0.834	0.457	0.597	0.313	0.348	0.256	0.111	0.276
C, 0 vs 1–4	0.802	0.788	0.779	0.531	0.684	0.687	0.652	0.744	0.889	0.724
C, 0, 1 vs 2–4	0.752	0.725	0.714	0.708	0.44	0.695	0.675	0.78	0.768	0.729
C, 0–2 vs 3, 4	0.772	0.707	0.68	0.7	0.461	0.661	0.758	0.929	0.778	0.831
C, 0–3 vs 4	0.781	0.705	0.69	0.716	0.429	0.543	0.897	0.947	0.851	0.958

As comparison of SOX9 target proteins performance, we compared all targets against previously validated biomarkers of fibrosis in the same samples. Specifically, hyaluronic acid (HA), Procollagen 3 N-peptide (P3NP), TIMP1, AST-to-Platelet Ratio Index (APRI) and the combined ELF panel were assayed and compared. All validated biomarkers/APRI values were taken at time of serum collection using unfrozen sample, in contrast to the experimental markers which were assessed in serum after going through a freeze-thaw cycle. Direct comparison of the AUROC values for each marker identified OPN and VIM as superior to APRI across all stages of fibrosis (Table 2). OPN and VIM also exhibited higher diagnostic performance at distinguishing earlier stages of fibrosis (controls and F0 fibrosis) compared to HA and P3NP, with TIMP1. However, HA, P3NP, TIMP and the combined ELF panel were superior in predicting F4/cirrhosis (Table 2).

## Discussion

We have previously described a critical role for SOX9 in the mechanisms underlying liver fibrosis^12–16^. Moreover, in biopsy samples from patients with chronic liver disease we have established SOX9 as a predictive marker of progression toward cirrhosis^12^. In this study, we applied our knowledge of SOX9 in matrix regulation to identify and profile a panel of downstream targets in serum samples from patients with varying stages of chronic liver disease. Our transcriptomic analysis of *Sox9* depleted HSCs revealed the extent of its role in regulating ECM components associated with fibrosis. Combined with our own data in liver fibrosis and studies in other organs^12–14,16,25,27–29^, we were able to further scrutinize these data to identify a cohort of highly expressed ECM proteins amenable to assay in patient serum samples, including OPN, VIM, SPARC, GPNMB and FN1. All targets were localized and highly expressed in activated HSCs from rodent and human. Four of the five targets had SOX9 binding sites in their promoter region with increased enrichment for SOX9, whereas VIM demonstrated comparative changes with *Sox9* knockdown suggesting indirect regulation by SOX9. *In vivo*, all targets were reduced following *Sox9* deletion in mouse models of fibrosis and localized to fibrotic regions in biopsy tissue from patients with severe fibrosis, commensurate with increased SOX9 levels.

Although liver biopsy remains the gold-standard to assess fibrosis in the case of diagnostic uncertainty, several non-invasive diagnostic tools now in clinical practice are based on mechanisms underlying the disease process. For example, physical approaches measuring liver stiffness, such as transient elastography (TE), take advantage of altered organ biomechanics in response to scarring; whereas many serum biomarker panels rely on detection of known ECM components indicative of the disease process^9,30,31^. Despite their widespread use to distinguish early/no fibrosis from cirrhosis, classifying patients with intermediate stages of fibrosis has proved challenging. Combined with our own insight into the role of SOX9 in fibrosis, we tested the SOX9-regulated ECM proteins as novel serum markers in a well phenotyped cohort of patients with variable fibrosis. Our data indicted two SOX9-regulated ECM proteins, OPN and VIM have potential as biomarkers of liver fibrosis severity; interestingly, the data, for instance on AUROCs, were comparable to those from previous studies scrutinising other validated individual biomarkers and panels (reviewed in^8^). Supporting OPN and VIM as dynamic biomarkers, the distribution of both factors became significantly reduced in regions of scarring in mouse models of fibrosis resolution^32,33^. Conversely, elevations of OPN have been implicated in the progression of multiple chronic liver diseases, associated with fibrosis, including non-alcoholic steatohepatitis, alcoholic liver disease, and infection with either HCV or HBV^20,21,34,35^.

Despite FN1 performing poorly as a biomarker for progressive fibrosis, our data in the experimental cohort complemented previous work highlighting its potential to predict early fibrosis^36^. Consistent with these findings, fibronectin isoforms are thought to precede α-SMA expression and be involved in myofibroblast activation by profibrotic TGF-β^37,38^. Similar results were seen between the SOX9-regulated ECM proteins and three established clinical biomarkers (including APRI). Although TIMP1, HA and P3NP were better at identifying cirrhosis; OPN and VIM demonstrated superiority at earlier stages of fibrosis. This proof of concept diagnostic study provides encouragement for these biomarkers to be tested in larger phase 3 diagnostic studies. Importantly, a combined panel of SOX9 regulated ECM proteins may show superior performance for identifying earlier stages of fibrosis.

Overall, these data further support a critical role for SOX9 in the mechanisms underlying fibrosis and indicate the value of investigating SOX9-regulated pathways as serum biomarkers or as potential targets to reduce fibrosis and its progression to cirrhosis and HCC. Significantly, in our previous study, the utility of SOX9 detection in biopsy samples to detect severity and predict disease progression outperformed all other fibrosis risk factors^12^. Collectively these current and previous data highlight a potential clinical use for SOX9 and its downstream secreted targets as a measure to stratify patients with liver fibrosis alongside existing or emerging measures (e.g. cirrhosis risk score or liquid biomarkers)^39–42^.

## Materials and Methods

### Human liver tissue and serum

Liver biopsies tissue used in this study has been described previously^12^. Briefly, paired biopsy samples were obtained with informed consent and ethical approval from the Trent Cohort Study of Hepatitis C Virus (HCV) antibody‐positive patients from across the former UK Trent Health Region^43,44^ following selection and data collection criteria as previously described^43–45^. Patients who were receiving therapy or infected with human immunodeficiency virus were excluded. Liver biopsies were assessed blindly by an expert liver histopathologist based on the 7-point Ishak fibrosis stage, IS0 to IS6; the latter representing the most severe fibrosis/cirrhosis^26^. We identified a cohort of 152 biopsies classified as mild fibrosis (IS0–1; n = 100) intermediate (IS2–3; n = 30) or severe disease (IS4–6; n = 22)^12^. From the same Trent HCV Cohort Study, we identified serum samples amenable to assay as a validation cohort. This consisted of 131 serum samples classified as IS0 (no fibrosis; n = 52), IS1–2 (mild fibrosis; n = 60) and IS5–6 (severe fibrosis/cirrhosis; n = 19).

As proof of concept of diagnostic performance we tested serum samples in an independent, external cohort of patients with chronic HCV (treatment naïve) and healthy controls (LREC no. 04/Q1701/58). All subjects were asked to follow dietary restrictions and avoid medicinal products for seven days prior to blood sampling and collection occurred between 9 am and 11am. All patients with HCV underwent a liver biopsy which was assessed by an expert liver histopathologist using the METAVIR scoring system. All biopsies were greater than 15 mm in length and contained more than six portal tracts. All research was performed in accordance with relevant guidelines and regulations.

### Animal models of liver fibrosis and treatments

All animal research was performed in accordance with relevant guidelines and regulations, as described previously^12,23^. Mice were housed and maintained, and animal experiments carried out, with approval from the University of Manchester Ethical Review Committee in accordance with UK Government Home Office regulations and its approval (licence P7FDDE62C). Mice were on a C57BL/6 J background in housing with a 12-hour light dark cycle and food and water available *ad libitum*. RosaCreER mice^12,46^ were sourced from Jackson Laboratories. *Sox9*^fl/fl^ mice were a kind gift from Professor Gerd Scherer^12,47^. To achieve inducible global *Sox9* deletion, *Sox9*^fl/fl^ mice were crossed with RosaCreER mice to generate RosaCreER:*Sox9*^fl/fl^ animals. Genotyping, fibrosis induction and gene inactivation by tamoxifen for animals used in this study has been published^12^. Briefly, tamoxifen (Sigma, UK) was injected i.p. to activate CreER activity and induce *Sox9* deletion in ROSACreER:*Sox9*^fl/fl^ animals. ROSACreER^+/−^ and ROSACreER^−/−^ animals were injected with tamoxifen to control for any unexpected effects. 8 week carbon tetrachloride injections (CCl_4_) and two week bile duct ligation (BDL) were used to induced fibrosis in mice. Tissue and serum samples were collected at the end of the procedure for analysis.

For both CCl_4_ and BDL models of fibrosis in RosaCreER:*Sox9*^fl/fl^ animals, there were four experimental groups. CCl_4_: RosaCreER^−/−^:*Sox9*^fl/fl^ Olive Oil (n = 6); RosaCreER^+/−^:*Sox9*^fl/fl^ Olive Oil (n = 6), RosaCreER^−/−^:*Sox9*^fl/fl^ CCl_4_ (n = 5), and RosaCreER^+/−^:*Sox9*^fl/fl^ CCl_4_ (n = 8). BDL: RosaCreER^−/−^:*Sox9*^fl/fl^ Control (n = 5); RosaCreER^+/−^:*Sox9*^fl/fl^ Control (n = 5), RosaCreER^−/−^:*Sox9*^fl/fl^ BDL (n = 7), and RosaCreER^+/−^:*Sox9*^fl/fl^ BDL (n = 5)^12^.

### Histology, immunohistochemistry and immunocytochemistry

Tissue samples were fixed in 4% paraformaldehyde (PFA) and processed for histology or immunohistochemistry (IHC) as described previously^12,23^. HSCs grown on chamber slides were also fixed in 4% PFA, then stored in PBS at 4 °C as for previous work^13,16^. For IHC in tissue, 10 mM sodium citrate (pH 6) was used for antigen retrieval, except for Osteopontin IHC which required pepsin (Sigma, UK). Antibodies used are listed in Supplementary Table 1. Histological collagen staining was carried out using picro-sirius red (PSR)^12,23^. All histological quantification and analysis was carried out blind from scanned slide images following histology as previously described^12^.

### Primary cell culture, Quantitative PCR, Western Blotting and microarray

Primary rat and human hepatic stellate cells (rHSCs) were isolated and RNA and protein prepared as described previously^13,16^. Gene silencing for SOX9 was carried out in culture activated HSCs using short interfering RNA (siRNA) as previously indicated. qPCR used intron spanning primers wherever possible (Supplementary Table 2). Western blotting was done following standard protocols^13,16^. Protein bands were detected with primary and secondary antibodies listed in Supplementary Table 1. For microarray, sample preparation and analysis are detailed in supplementary methods^23^.

### Elisa

Two variations of the ELISA protocol were used due to the availability and applicability of antibodies. Sandwich ELISA, whereby a coating antibody was initially used to capture the antigen prior to a detecting antibody being used to detect the relative concentration by comparing to the optical density at 450 nm of known standards. Where suitable capture antibodies were not available, direct ELISA was utilized and the serum sample was coated directly onto the plastic wells and a detecting antibody used to assay. A dilution series of known concentration recombinant human proteins (R&D systems) were used as standards. Accuracy of ELISAs was measured with coefficients of variation for each marker assayed and a sample was discounted if variation was >10% between replicates. Antibodies used are shown in Supplementary Table 1.

### Chromatin Immunopreciptation

Conserved SOX9 binding sites were identified using ECR browser (http://ecrbrowser.dcode.org) and the MUltiple sequence Local AligNment and conservation visualization tool (MULAN: https://mulan.dcode.org/). Chromatin immunoprecipitation (ChIP) assays were performed as described previously^16^. Following chromatin isolation and immunoprecipitation with a SOX9 antibody (sc-20095, H-90, Santa Cruz), protein-DNA complexes were eluted, crosslinks reversed and protein degraded prior to DNA purification and PCR (Supplementary Table 1).

### Statistical Analysis

Data was analysed using the SPSS 19 (IBM, USA) software package in multiple samples after a minimum of 3 determinations. Where appropriate, data was expressed as mean ± standard error of the mean (SEM). In samples with 2 groups, comparison was made using an un-paired *t*-test, assuming unequal variance. For ELISA, assay consistency and accuracy was assessed using the average coefficient of variation (CoV). Serum markers’ ability to discriminate between stages of fibrosis was achieved by area under the receiver operator curve (AUROC) analysis with 95% confidence intervals stated.

## Electronic supplementary material

Supplementary Information

Supplementary dataset 2

Supplementary dataset 1
