# Publisher Correction: SOX9 regulated matrix proteins are increased in patients serum and correlate with severity of liver fibrosis

**DOI:** 10.1038/s41598-019-47715-2

**Published:** 2019-08-06

**Authors:** Varinder S. Athwal, James Pritchett, Katherine Martin, Jessica Llewellyn, Jennifer Scott, Emma Harvey, Abed M. Zaitoun, Aoibheann F. Mullan, Leo A. H. Zeef, Scott L. Friedman, William L. Irving, Neil A. Hanley, Indra N. Guha, Karen Piper Hanley

**Affiliations:** 1 Wellcome Centre for Cell-Matrix Research, Faculty of Biology, Medicine & Health, Manchester Academic Health Science Centre, University of Manchester, Oxford Road, Manchester, M13 9PT UK; 2 Division of Diabetes, Endocrinology and Gastroenterology, Faculty of Biology, Medicine & Health, University of Manchester, Manchester Academic Health Science Centre, Oxford Road, Manchester, UK; 3 Research & Innovation Division, Central Manchester University Hospitals NHS Foundation Trust, Oxford Road, Manchester, M13 9PT UK; 4 School of Healthcare Science, Manchester Metropolitan University, Manchester, M1 5GD UK; 5 Department of Cellular Pathology, NIHR Biomedical Research Centre, Nottingham University Hospitals NHS Trust and University of Nottingham, Nottingham, UK; 6 Bioinformatics Core Facility, Faculty of Life Sciences, University of Manchester, Manchester, UK; 7 Division of Liver Diseases, Icahn School of Medicine at Mount Sinai, New York, NY10029 USA; 8 NIHR Biomedical Research Centre, Nottingham University Hospitals NHS Trust and University of Nottingham, Nottingham, UK; 9 School of Life Sciences, NIHR Biomedical Research Centre, Nottingham University Hospitals NHS Trust and University of Nottingham, Nottingham, UK

Correction to: *Scientific Reports* 10.1038/s41598-018-36037-4, published online 17 December 2018

In the original version of this Article, the author Karen Piper Hanley was incorrectly indexed. This error has now been corrected.

