# Supplementary material for: SOX9 regulated matrix proteins are increased in patients serum and correlate with severity of liver fibrosis
Source: Sci Rep. 2018 Dec 17;8:17905. doi: 10.1038/s41598-018-36037-4 (PMC6297163; doi:10.1038/s41598-018-36037-4)
Supplement: Supplementary file 1 — Supplementary Information [file 41598_2018_36037_MOESM1_ESM.pdf]

Supplementary Information

SOX9 regulated matrix proteins are increased in patients serum and correlate with severity of liver fibrosis

Varinder S Athwal [1,2,3,10], James Pritchett [1,2,4,10], Katherine Martin [1,2,3], Jessica Llewellyn [2], Jennifer Scott [1,2,3], Emma Harvey [2], Abed M Zaitoun [5], Aoibheann F Mullan [1,2,3], Leo A H Zeef [6], Scott L Friedman[7], William L Irving [8, 9], Neil A Hanley [2,3], Indra N Guha [8] & Karen Piper Hanley [1,2,3*].

[1] Wellcome Centre for Cell-Matrix Research, Faculty of Biology, Medicine & Health, Manchester Academic Health Science Centre, University of Manchester, Oxford Road, Manchester M13 9PT, UK; [2] Division of Diabetes, Endocrinology and Gastroenterology, Faculty of Biology, Medicine & Health, University of Manchester, Manchester Academic Health Science Centre, Oxford Road, Manchester, UK; [3] Research & Innovation Division, Central Manchester University Hospitals NHS Foundation Trust, Oxford Road, Manchester, M13 9PT, UK; [4] School of Healthcare Science, Manchester Metropolitan University, Manchester, M1 5GD, UK. [5] Department of Cellular Pathology, NIHR Biomedical Research Centre, Nottingham University Hospitals NHS Trust and University of Nottingham, Nottingham, UK; [6] Bioinformatics Core Facility, Faculty of Life Sciences, University of Manchester, Manchester, UK; [7] Division of Liver Diseases, Icahn School of Medicine at Mount Sinai, New York, NY10029, USA; [8] NIHR Biomedical Research Centre, Nottingham University Hospitals NHS Trust and University of Nottingham, Nottingham, UK; [9] School of Life Sciences, NIHR Biomedical Research Centre, Nottingham University Hospitals NHS Trust and University of Nottingham, Nottingham, UK.

[8] Both authors contributed equally to this manuscript.

* Corresponding author: karen.piperhanley@manchester.ac.uk

A

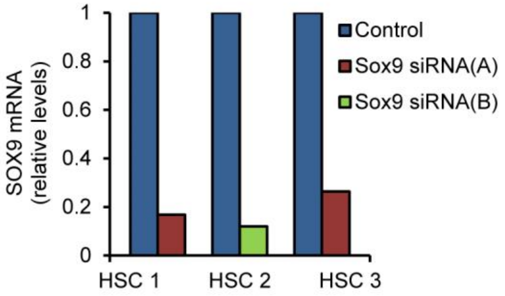

B

Effect of Sox9 siRNA	Gene expression ≥ 1.2 fold (P<0.05)
**upregulated**	250
**downregulated**	290

C

D

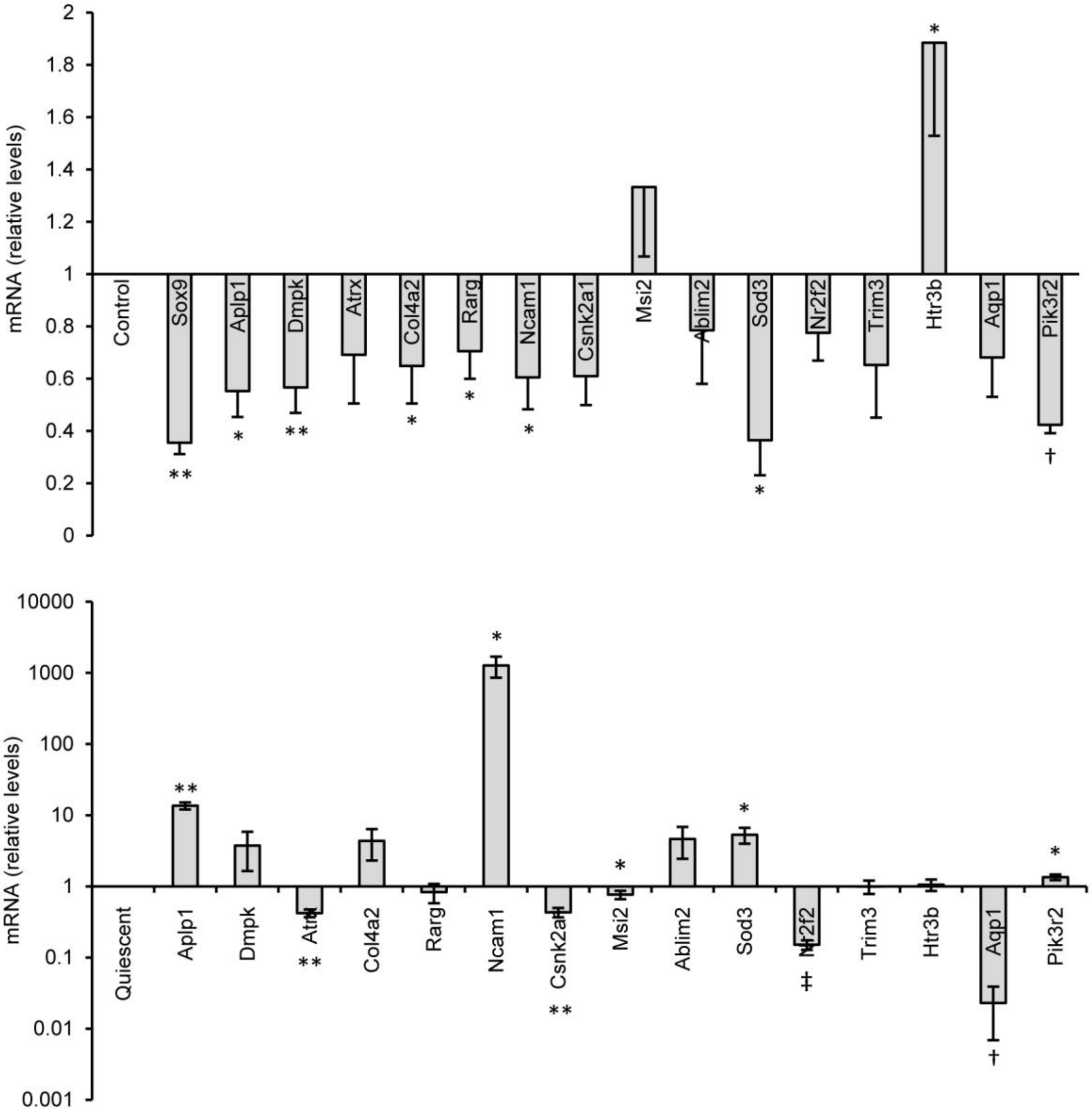

Supplementary Figure 1. (A) Sox9 mRNA levels in samples sent for microarray analysis. Activated rat HSCs from 3 different animals (HSC1-3) shown with Sox9 knockdown by two different siRNAs (siRNA A & B) compared to scrambled control. (B) Summary of differentially expressed genes resulting from Sox9 abrogation as detected by the microarray. (C) mRNA verification of top 15 positively regulated SOX9 genes detected by the microarray (i.e. downregulated in array) in HSCs following Sox9 knockdown and in quiescent and activated rat HSCs. Data are shown as means ± s.e.m. *P<0.05, **P<0.01, †P<0.005, ‡P<0.001; n=3.

CLUSTER	CORE CATAGORY	P VALUE	GENE SYMBOL
**Extracellular Matrix (ECM)<br>Functional Annotation Clustering - Combined Enrichment Score (ES) = 10.15**			
ECM region<br>(ES = 4.18)	Signal peptide<br><br>Extracellular region<br><br>Glycoprotein<br><br>Disulfide bond	2.3E-6<br><br>3.2E-6<br><br>1.0E-5<br><br>2.1E-4	*Htr3b, Abca2, Abcc5, Atp2a2, Atp6ap2, Clecsf6, Cd109, Cd200r1, Cd9, Cd2Cd53, Frem3, Fcgrt, Gpr6, G7c, RT1-A3 A3, St3gal6, Thy1, Wisp2, Actn4, Actn1, Aoc3, Angptl4, Aqp1, Bmp3, Clstn2, Car4, Ceacam1, Ctsk, Ccl24, Ccr5, Cxcl13, Cxcl14, Cxcl2, Cxcl6, Chid1, Ch25h, Chrm1, Cspg4, Nptxr, Col1a1, Col4a1, Col4a2, C1qa, Cfd, Cxadr, Dci, Ednra, Fam174a, Fgf18, Fgf2, Flt1, Fzd2, Fut11, Gabrb1, Gabbr2, Gpx3, Hyal3, Ecm1, Hyou1, Igfbp5, Itgbl1, Il1m, Kalrn, Kremen1, Lama2, Lphn1, Lphn2, Lrrc33, Lmf2, Lbp, Lamp1, Mmp3, Mmp7, Mgea5, Muc10, Ncr3, Ncam1, Nrg1, Nxph1, Nbl1, Ncan, Nrgn, Nmu, Nrcam, Npy, Npy1r, Nrp1, Ncstn, Nucb1, Nup155, Prph2, Plaur, Pnoc, Prl6a1, Prl7a3, Prl8a9, Prelp, Prap1, Pcsk1, Ptger2, Prss8, P2ry12, Ramp2, Sparc, Sctr, Srgn, Serpina3k, Serpini1, Srprb, Scnn1a, Slc1a3, Slc22a4, Slc28a1, Slc34a1, Slc38a4, Slc7a1, Slco1a4, Sstr1, Sort1, S1pr5, Sod3, Sftpa1, Sftpb, Tln1, Trhr, Tgfb3, Tmem150, Vipr1, Vcan, Wnt2, Zfhx4*
Bone<br>(ES = 3.09)	Skeletal system development	6.4E-4	*Pdlim7, Bmp3, Ctsk, Col1a1, Dlk1, Fgf18, Fgfr2, Hoxd3, Igfbp5, Men1, Ptger2, Prkca, Rarg, Sparc, Sort1, Sgpl1, Tcfap2a, Tgfb3*
ECM part<br>(ES =2.88)	ECM	7.3E-5	*Frem3, Angptl4, Cspg4, Col1a1, Col4a1, Col4a2, Ecm1, Lama2, Mmp3, Mmp7, Ncan, Prelp, Sparc, Srgn, Srprb, Slc1a3, Sod3, Sftpa1, Tgfb3, Vcan, Wnt2*
**Vasculature<br>Functional Annotation Clustering - Combined Enrichment Score (ES) = 6.62**			
Vessel Development<br>(ES = 3.79)	Tube development<br><br>Blood vessel development	2.8E-2<br><br>2.4E-4	Gata6, Smarca4, Thy1, Angptl4, Cxcr4, Cspg4, Col1a1, Csrp3, Ednra, Fgf18, Fgf2, Fgfr2, Flt1, Hand2, Myo1e, Nrp1, Nr2f2, Sparc, Sgpl1, Tcfap2a, Tgfb3, Ubp1, Wnt2, Zeb2, Zfpm2
Circulation<br>(ES = 2.83)	Blood circulation	5.3E-4	Atp6ap2, Gch1, Acta2, Cxcr4, Cyp11b1/b2, Ednra, Mecp2, Nrg1, Npy, Npy1r, Pde5a, Scnn1a, Tpm1
**Signalling<br>Functional Annotation Clustering - Enrichment Score (ES) = 4.2**			
Hormones<br>(ES = 2.2)	Response to organic substrate	1.1E-2	Pfkfb2, Abca2, Abcc5, Atp2a2, Fosb /// Fosl2, Gch1, Acta2, Adfp, Aqp1, Car4, Ccr1, Ccr5, Col1a1, Cdkn1a, Cyp2d1 /// Cyp2d5, Ednra, Gpx3, Il1rn, Irak2, Lbp, Lrp5, Mmp3, Mgea5, Npy1r, Pcsk1, Prss8, Prkca, Ramp2, Rcan1, Sparc, Srprb, Stat3, Stat6, Prkaca, Sort1, Tbl1x, Tgfb3
Corticoids<br>(ES = 2.0)	Response to drug<br><br>Response to organic cyclic substrate<br><br>Response to corticosteroid stimulus	5.2E-2<br><br>1.9E-2<br><br>1.3E-2	Fosb /// Fosl2, Acta2, Adfp, Car4, Col1a1, Cdkn1a, Cyp2d1 /// Cyp2d5, Cyp11b1 /// Cyp11b2, Ednra, Fosb /// Fosl2, Gpx3, Il1rn, Pde4a, Pols, Pcsk1, Prss8, Prkca, Sparc, Srprb, Stat3, Slc1a3
**Cytoskeleton<br>Functional Annotation Clustering - Enrichment Score (ES) = 2.71**			
Cytoskeleton	Arrhythmogenic right ventricular cardiomyophathy<br><br>Regulation of actin cytoskeleton<br><br>Focal adhesion	6.6E-5<br><br>5.2E-2<br><br>2.2E-3	Atp2a2, Shc2, Actn3, Actn4, Actn1, Chrm1, Col1a1, Col4a1, Col4a2, Fgf18, Fgf2, Fgfr2, Flt1, Itga3, Itga9, Itga11, Lama2, Pip5k1a, Pik3r2, Pkp2, Prkca, Tln1, Atf3
**Lysosome<br>Functional Annotation Clustering - Enrichment Score (ES) = 2.04**			
Lysosome	Vacuole	8.9E-4	Abca2, Atp6v1g2, Rab14, Ctsk, Chid1, Cit, Hyal3, Laptm5, Lamp1, Ncstn, Srgn, Sort1, Sult1c2, Sftpb
**Transcriptional Regulation<br>Functional Annotation Clustering - Enrichment Score (ES) = 2.02**			
Transcriptional regulation	Positive regulation of macromolecule metabolic process<br><br>Transcription factor binding<br><br>Regulation of RNA metabolic process<br><br>Regulation of transcription	3.4E-2<br><br>3.0E-3<br><br>2.7E-3<br><br>2.0E-2	Apex1, Abca2, Fbxo5, Fos, Fosb, Fusip1, Gata6, Gatad2b, Garnl1, Khsrp, Klf9, Sep-6, Rasd1, Satb1, Setd8, Sox4, Smarca4, Thy1, Ablim2, Afap1l2, Atf3, Ahr, Bmp3, Bmyc, Crem, Calcoco1, Creg1, Cbx1, Clock, Cdk9, Ednra, Fgf2, Fosl2, Hand2, Hsf2, Hoxd3, Itga9, Irf2, Irak2, Irak4, Med13, Mgea5, Mecp2, Men1, Nrg1, Nfe2, Nr2f2, Psmd14, Prkca, Rarg, Stk4, Stat3, Stat6,Sgsm3, Sorbs3, Tcf3, Tcfap2a, Tcfcp2l1, Tbl1x, Tgfb3, Usf2, Zeb2, Zbtb24, Zfhx2, Zfhx4, Zfp36l2, Znf597, Znf819, Zfpm2, Zhx3

Supplementary Figure 2. Functional annotation clustering. Individual categories and Sox9-regulated genes underlying them are shown. Enrichment Score >2.0.

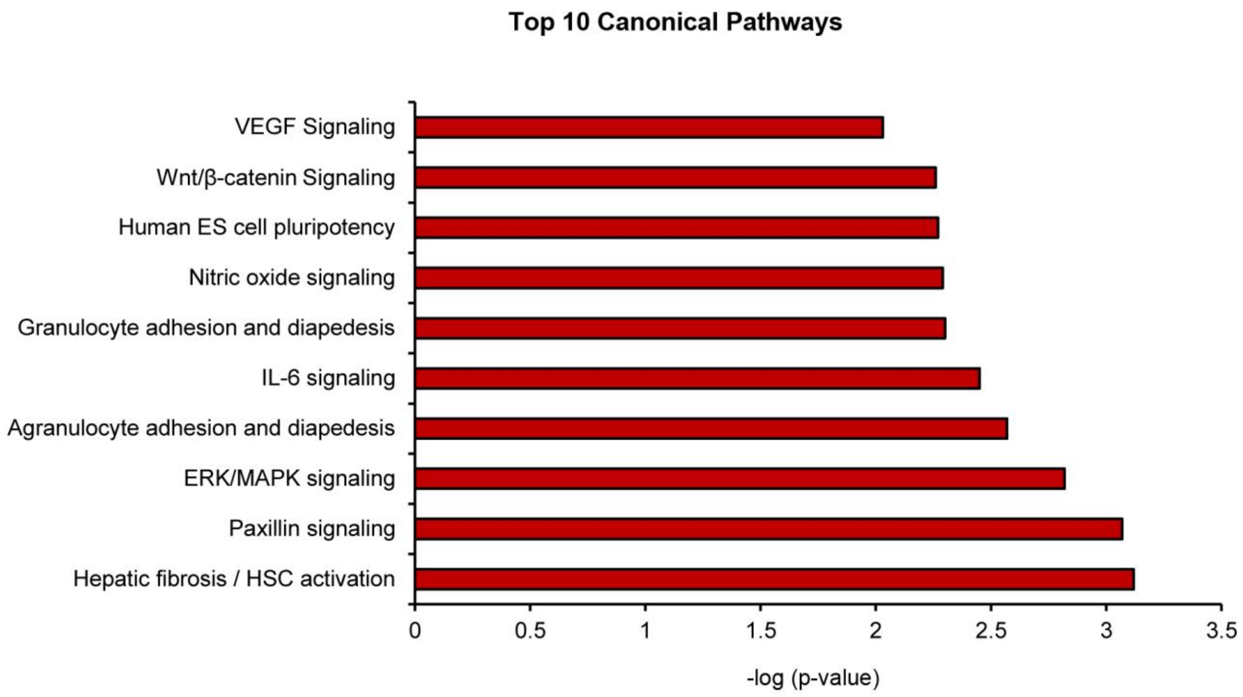

Supplementary Figure 3. Top 10 canonical pathways of differentially expressed Sox9-regulated genes following Ingenuity Pathway Analysis. Pathways were ranked by the negative log of P-values calculated by Fisher's exact test for gene enrichment.

FN1

```
Human   TTTATATTTCAATTGTTATTTACTCCAAGAGTGTGGAAAGTTCTCCTTTT
Mouse   TTTATATTTCAATTGTTATCTGCTCCAAGAATGTATAAAGTTCCACTGAT
Rat     TTTAGATTTCAATTGTTATCTGCTCCAAGAATGTATAAAGTTCCACTGAT
        **** ************** * ******** ***  *******  **  *
```

GPNMB

```
Human   AGGATTCAGGGGCAGAGAACTTGAATCAATGGGGTCTTTTTATTTTTGAACA
Mouse   AGGACTCAAGGGCTGAGAACCTGGATCAATGGAGTC--TTTATTTTGGAACA
Rat     AGGACTCAGGGGCCGAGAACTTGGATCAATGGGGTC--TTTATTTTTGAACA
        **** *** **** ****** ** ******** ***  ******** *****
```

SPARC

```
Human   CCAGCAAGCT-GAGCAAATCCCTGAGAACAAAAGGGGGAGAAGACCTG-ACAA
Mouse   CCAGTAAGCAGGAGCAGATCCCAGAGAAC-AAAGGGAGGAGAGGACTCC---A
Rat     CCAGCAGGCA-AAGCAGATCCCTGAGAAC-AAAGGGAGGAGAGGACTCC-AGA
        **** * **   **** ***** ****** ****** *   **  **     *
```

VIM

```
Human   TTCCAATCTCAGGCGCTCTTTGTTTCTTTCTCCGCGACTTCAGATCTGAGG
Mouse   CCCCAATCTCGGGCCCTCTTTGTTTCTCTCTCCCCGGCTTCCGATCTGCAG
Rat     CCCCAATCTCAGGCCCTCTTTGTTTCTCTCTCCCCGCCTTCCGATCTGCAA
          ******** *** ************ ***** ** **** ******
```

Supplementary Figure 4. Alignment of upstream enhancer regions with conserved SOX9-binding motif highlighted in black. Conserved nucleotides indicated by asterisk (*). The core SOX-binding motif is CAAT with increased binding affinity for SOX9 conferred by additional flanking nucleotides (1). The SOX9-binding region for OPN has been previously characterised and described (2).

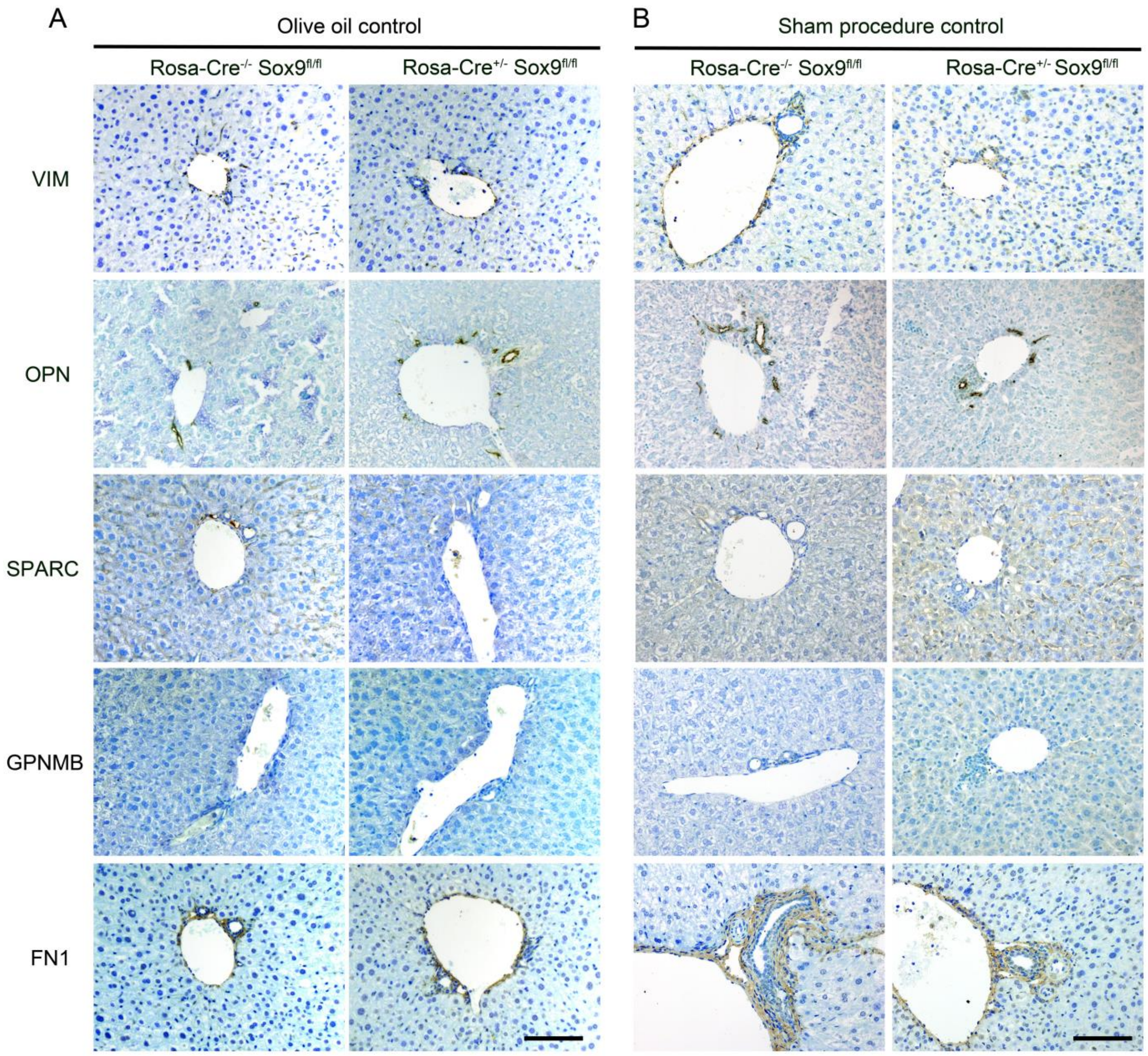

Supplementary Figure 5. Immunohistochemistry for VIM, OPN, SPARC, GPNMB and FN1 (brown) in olive oil (A) and sham operative procedure (B) as non-fibrotic control tissue for fibrosis induction by CCl_4 and BDL respectively. Tissue sections are from Sox9-control animals (RosaCre-/-; Sox9fl/fl) and Sox9-null animals (RosaCre+/-; Sox9fl/fl) for data shown in Figures 4 and 5.

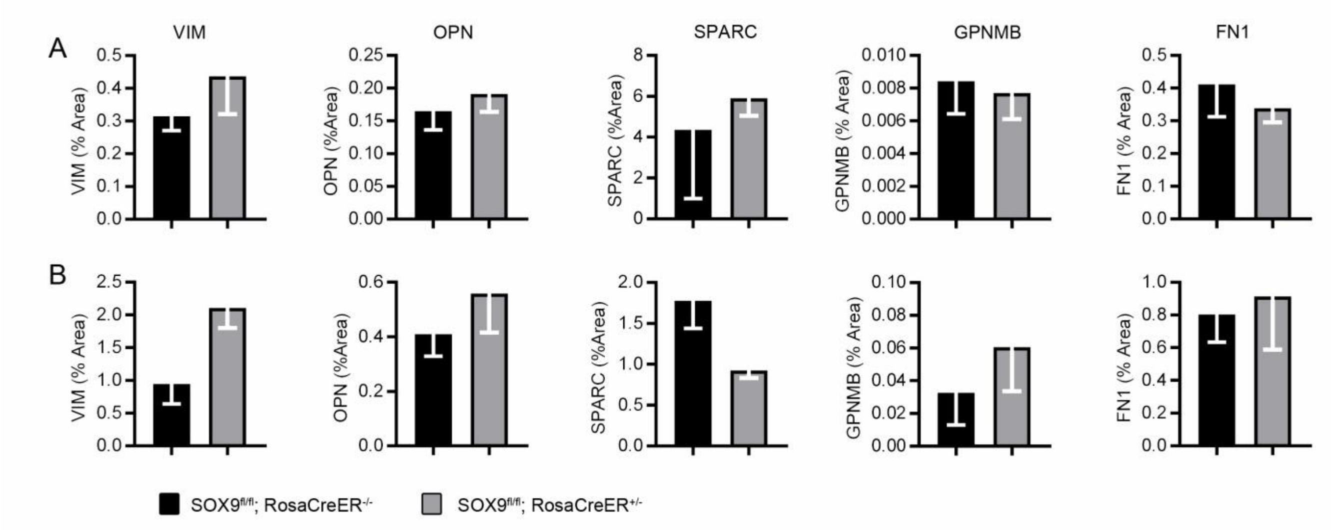

Supplementary Figure 6. Quantification of SOX9-regulated proteins in control liver for Supplementary Figure 5. A. Olive oil administered as control for CCl_4. B. Sham procedure for BDL. A-B. Quantification of surface area covered by individual protein staining in control (Sox9fl/fl; RosaCreER-/-) and *Sox9*-null (Sox9fl/fl; RosaCreER+/-) livers. All experiments are n = 5. Data are shown as means ± s.e.m.

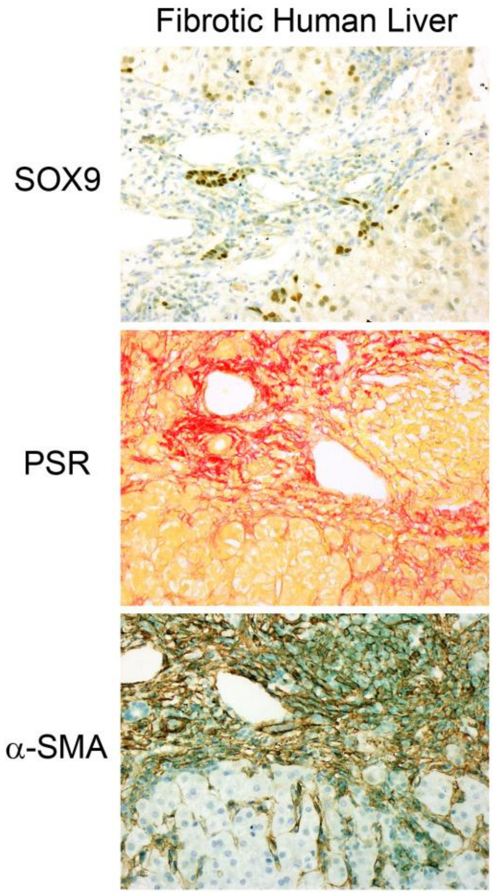

Supplementary Figure 7. Example serial tissue sections from a severe fibrotic/cirrhotic patient biopsy sample (IS 6) showing extent of SOX9 and α-SMA localization by immunohistochemistry (brown). Fibrosis is histologically localized by picrosirius red (PSR) staining (collagen deposition in red). Quantification of SOX9 in severity and progression of fibrosis has been described for these samples (3).

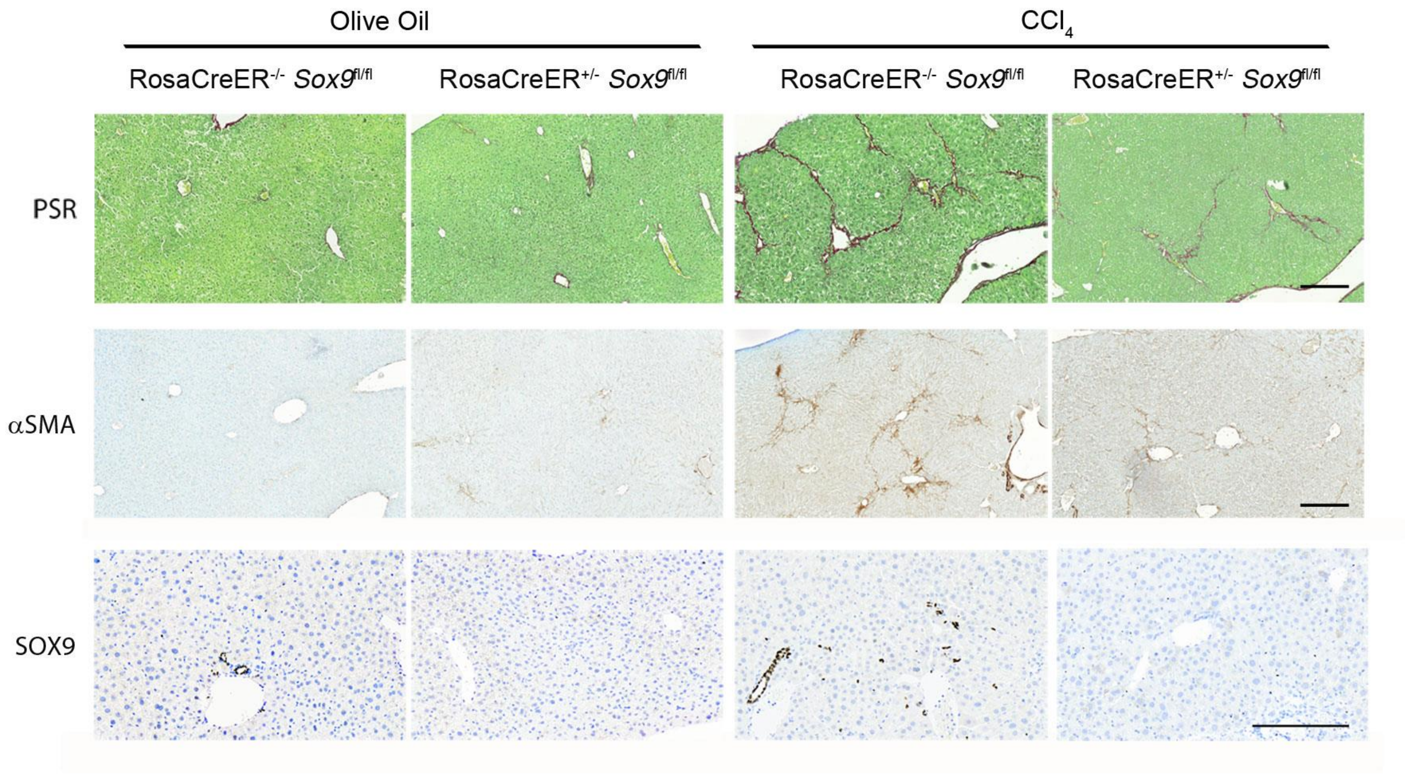

Supplementary Figure 8. Example tissue sections showing; picrosirius red (PSR) staining (collagen deposition in red) counterstained with fast green (top row) and immunohistochemistry for α-SMA (brown staining middle row; activated HSC / myofibroblast marker) and SOX9 (brown) in olive oil treated (left) or chronic CCl_4 induced fibrosis (right) in control and Sox9-null mice. Size bar = 200 μm. Liver function, body weight and quantification data has been described for this model previously (3).

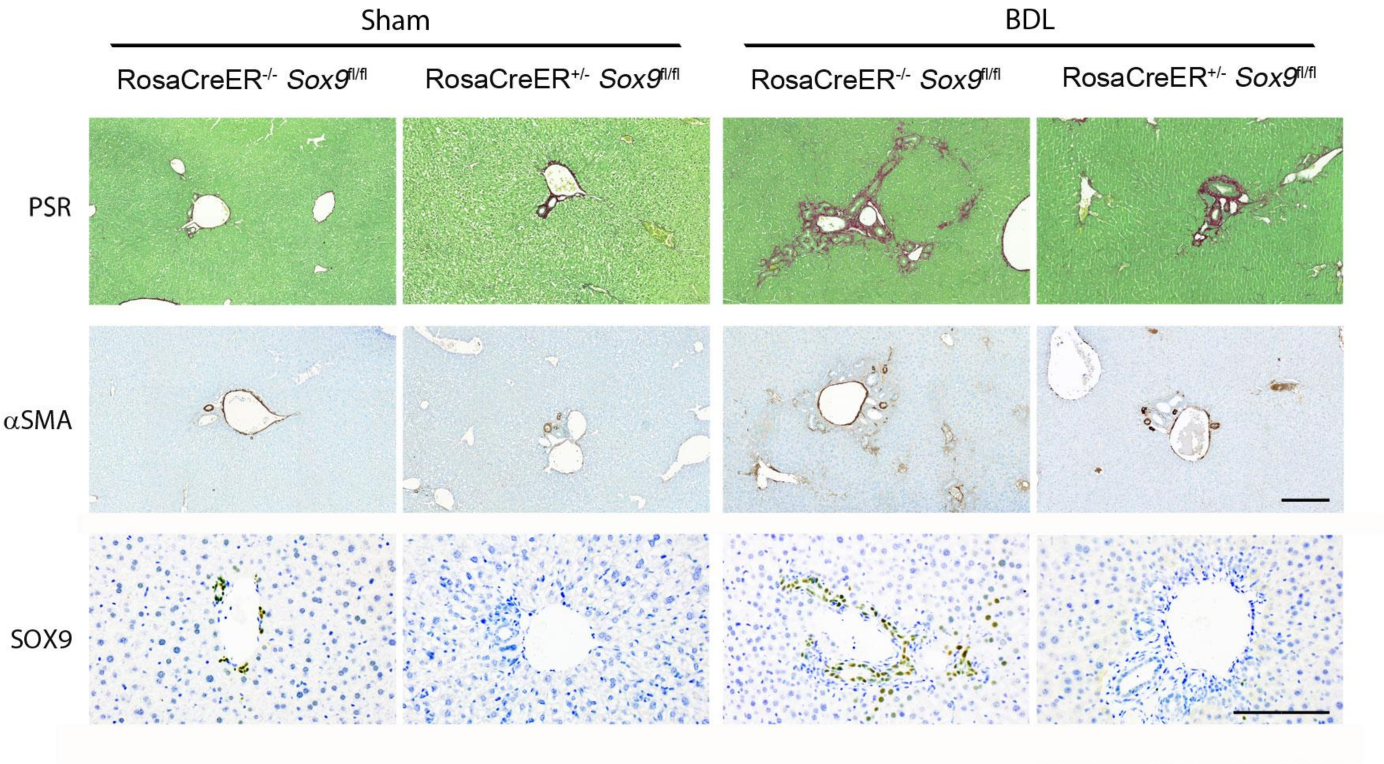

Supplementary Figure 9. Example tissue sections showing; picrosirius red (PSR) staining (collagen deposition in red) counterstained with fast green (top row) and immunohistochemistry for α-SMA (brown staining middle row; activated HSC / myofibroblast marker) and SOX9 (brown) in sham (left) or BDL induced fibrosis (right) in control and Sox9-null mice. Size bar = 200 μm. Liver function, body weight and quantification data has been described for this model previously (3).

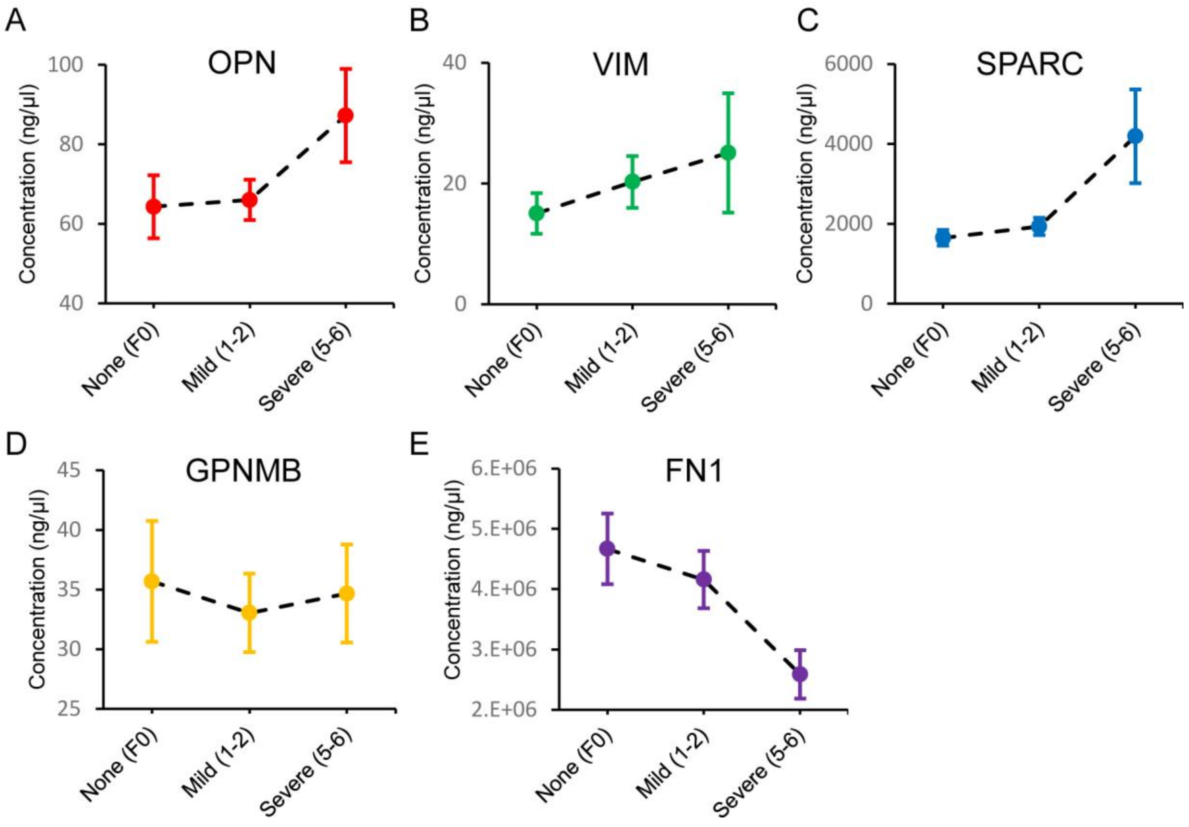

Supplementary Figure 10. SOX9-regulated proteins are present and increased in serum from patients with liver fibrosis. (A-E) Serum concentration of SOX9-regulated targets quantified by ELISA and grouped by stage of fibrosis (Ishak). Data are shown as means ± s.e.m.

Antibody	Raised In	Company	Dilution	Application
SOX9	Rabbit	Millipore	1:5000	Western
			1:800	IHC / ICC
SOX9	Rabbit	Santa Cruz	6μg/ml	ChIP
OPN	Goat	Abcam	1:750	Western
OPN	Mouse	Novocastra	1:50	ICC
OPN	Mouse	R&D Systems (MAB14332)	1:250	ELISA capture
OPN	Goat - biotinylated	R&D Systems	1:500	ELISA detect
β-actin	Mouse	Sigma	1:100 000	Western
Vimentin	Mouse	Novocastra	1:1000	ELISA detect
SPARC	Mouse	Novocastra	1:80	IHC / IF
SPARC	Goat	R&D Systems (AF941)	1:200	Western
SPARC	Mouse	R&D Systems (MAB941)	1:500	ELISA detect
FN1	Mouse	Abcam	1:1000	ELISA capture
			1:1000	Western
FN1	Rabbit	Abcam	1:2000	ELISA detect
			1:1000	ICC / IF
α-Enolase	Rabbit	Abcam	1:250	Western
GPNMB	Goat	R&D Systems (AF2550)	1:200	ELISA capture
GPNMB	Mouse	R&D Systems (MAB25501)	1:500	ELISA detect
Anti-mouse Alexafluor 488	Goat	Invitrogen	1:1000	ICC / IF
Anti-goat Alexafluor 488	Donkey	Invitrogen	1:1000	ICC / IF
Anti-rabbit Alexafluor 594	Donkey	Invitrogen	1:1000	ICC / IF

Supplementary Table 1. List of antibodies used with dilutions and application.

Supplementary Table 2. PCR primers used (for qPCR unless stated).

Gene	Species	Forward Primer	Reverse Primer
Cathepsin B (Ctsb)	**Rat**	CAACATGGCAGGCTGGACGCA	ACCCTTTCCGGCAGCTTGGGT
Cathepsin L1 (CtsL1)	**Rat**	ACGCCTTCGGTGACATGACCA	CCCTTCTTGTGCTTCTGGTGGCG
Cd63	**Rat**	ACCCAGGGCTGCGTGGAAAC	AGCAATGCCCAGGGCTGCTC
Chemokine (C-C motif) ligand 7 (Ccl7)	**Rat**	CATGCAGATCTCTGCCGCGC	ATTGGTCCCATCTGGTTGGGCCC
Connective tissue growth factor (Ctgf)	**Rat**	ATCCCTGCGACCCACACAAG	CAACTGCTTTGGAAGGACTCGC
Cox2	**Rat**	GCGCTCAGCCATGCAGCAAAT	CACCGTAGAATCCAGTCCGGGT
Crysatllin, alpha B (Cryab)	**Rat**	AGCGCCAGGACGAACATGGCTT	AGGATCCACGTCGGCTGGGATC
Enolase 1, alpha, transcript variant 1 (Eno1v1)	**Rat**	ATCAGCAAGGTCGTGGGCCG	TGAAGTTTCCGAGTCCTACACGCAC
Enolase 1, alpha, transcript variant 2 (Eno1v2)	**Rat**	TCTCTTCCGTGCTGCGGTGC	AGCCTTTGAGACACCCTTCCCCA
Fibronectin 1 (Fn1)	**Rat**	TGACCCCATCGACCAGTGCCA	CCCCAATGCCACGGCCGTAA
Fibronectin 1 (Fn1)	**Rat (ChIP)**	CCCTCGCCGCCAGATTTTT	ACGTGTTAGAGCTGTGAGCTT
Gapdh	**Rat**	GCACAGTCAAGGCCGAGAAT	GCCTTCTCCATGGTGGTGAA
Glycoprotein transmembrane nmb (Gpnmb, osteoactivin)	**Rat**	AAAGGGGCCACTCCCACGGAA	CGGGCTGCACACCCTGTTCT
Glycoprotein transmembrane nmb (Gpnmb, osteoactivin)	**Rat (ChIP)**	TTTTGCAGGCCCTCCCTTT	TAATGCTGGCTGGAAACCACA
GusB	**Rat**	CTCTGGTGGCCTTACCTGAT	AGGTGTTGTCATCGTCACCTC
Matrix Gla protein (Mgp)	**Rat**	AGTCAGTCCCTTCACCACCCGG	GTTCCCGGACTCTTTCCTGGGCT
MMP2	**Rat**	TTTGCTCGGGCCTTAAAAGTAT	CCATCAAACGGGTATCCATCTC
Osteopontin (Opn)	**Rat**	GAGTTTGGCAGCTCAGAGGA	TCTGCTTCTGAGATGGGTCA
Secreted protein, acidic, cysteine-rich (Sparc / osteonectin)	**Rat**	TTCCCAGCACCATGAGGGCC	AGCTTCCGTCTGAGGCGCTG
Secreted protein, acidic, cysteine-rich (Sparc / osteonectin)	**Rat (ChIP)**	ATGGCTCCCAGCCCAGTATT	AGTGCGGAAGGGGTTAAAGT
Sox9	**Rat**	GCAAGTAGCCCTGGTTTCGTTCTC	GGGTGGCCAGTGCTCAGTTGC
Thioredoxin 1 (Txn)	**Rat**	TGACTGCCAGGATGTTGCTGCA	AGCACCAGAGAACTCCCCAACCT
Transgelin (Tagln)	**Rat**	GCCCACAAACGACCAAGCCTTTTCT	TGCACTTCACGGCTCATGCCA
Tropomyosin 2 (Tpm2)	**Rat**	CTGGAAGAGACTTTGGCTAGTGCC	TCTGGTCGGCAGGTAGCAGGG
Vimentrin (Vim)	**Rat**	GGAGATGAGGGAGTTGCGCCG	CCTCCTGCAATTTTTCTCGCAGCCG
Vimentin (Vim)	**Rat (ChIP)**	ATCTTGGGCTGTGTGCTTCT	CGGTGTTGCAGATCGGAAGG
β-actin	**Rat**	CCCGCGAGTACAACCTTCT	CGTCATCCATGGCGAACT

Supplementary Table 3. Transcript gene expression (>3,800 arbitrary units) from microarray following ribosomal gene removal.

Gene Symbol	Gene Title
**Fth1**	ferritin, heavy polypeptide 1
**Vim**	vimentin
**Actb**	actin, beta
**Actb**	actin, beta
**Spp1**	secreted phosphoprotein 1
**Actg1**	actin, gamma 1
**Ctgf**	connective tissue growth factor
**Tmsb4x**	thymosin beta 4, X-linked
**Gpnmb**	glycoprotein (transmembrane) nmb
**COX1**	cytochrome c oxidase subunit 1
**Eef1a1 /// RGD1566344**	eukaryotic translation elongation factor 1 alpha 1 /// similar to eukaryotic tra
**S100a6**	S100 calcium binding protein A6
**Ccl2**	chemokine (C-C motif) ligand 2
**Tpm2**	tropomyosin 2
**Gapdh /// Gapdh-ps2**	glyceraldehyde-3-phosphate dehydrogenase /// glyceraldehyde-3-phosphate dehydrog
**Txn1**	thioredoxin 1
**Tpt1**	tumor protein, translationally-controlled 1
**Prdx5**	peroxiredoxin 5
**CYTB**	cytochrome b
**COX2**	COXII
**Ctsb**	cathepsin B
**Ctsl1**	cathepsin L1
**B2m**	beta-2 microglobulin
**Tagln**	transgelin
**S100a4**	S100 calcium-binding protein A4
**ND3**	NADH dehydrogenase subunit 3
**Lgals3**	lectin, galactoside-binding, soluble, 3
**Ldha**	lactate dehydrogenase A
**Ccl7**	chemokine (C-C motif) ligand 7
**S100a11**	S100 calcium binding protein A11 (calizzarin)
**Ppia**	peptidylprolyl isomerase A (cyclophilin A)
**Pf4**	platelet factor 4
**Eef1a1**	eukaryotic translation elongation factor 1 alpha 1
**Anxa2**	annexin A2
**Cd63**	Cd63 molecule
**Col1a2**	collagen, type I, alpha 2
**Hspa8**	heat shock protein 8
**Pabpc1**	poly(A) binding protein, cytoplasmic 1
**Cryab**	crystallin, alpha B
**Mgp**	matrix Gla protein
**Ftl**	ferritin, light polypeptide
**Aldoa**	aldolase A, fructose-bisphosphate
**Sparc**	secreted protein, acidic, cysteine-rich (osteonectin)
**Tuba1a /// Tuba1b /// Tuba1c**	tubulin, alpha 1A /// tubulin, alpha 1B /// tubulin, alpha 1C
**Myl6 /// Myl6b /// Myl6l**	myosin, light chain 6, alkali, smooth muscle and non-muscle /// myosin, light ch

**Lgals1**	lectin, galactoside-binding, soluble, 1
**Col1a2**	collagen, type I, alpha 2
**Ubb**	ubiquitin B
**Fau**	Finkel-Biskis-Reilly murine sarcoma virus (FBR-MuSV) ubiquitously expressed
**Tpm4**	Tropomyosin 4
**Pgam1**	phosphoglycerate mutase 1 (brain)
**Calm1**	calmodulin 1
**Fcgr2a /// LOC498276**	Fc fragment of IgG, low affinity IIa, receptor (CD32) /// Fc gamma receptor II b
**Eif1**	eukaryotic translation initiation factor 1
**Lyz2**	lysozyme 2
**Lox**	lysyl oxidase
**Fn1**	fibronectin 1
**Timp1**	TIMP metallopeptidase inhibitor 1
**RGD1309537**	similar to Myosin regulatory light chain 2-A, smooth muscle isoform (Myosin RLC-
**Ybx1**	Y box binding protein 1
**S100a10**	S100 calcium binding protein A10
**Col3a1**	collagen, type III, alpha 1
**Tagln2**	transgelin 2
**Gpx1**	glutathione peroxidase 1
**Timp2**	TIMP metallopeptidase inhibitor 2
**Eno1**	enolase 1, (alpha)
**Basp1**	brain abundant, membrane attached signal protein 1
**Gnb2l1**	guanine nucleotide binding protein (G protein), beta polypeptide 2 like 1
**P4hb**	prolyl 4-hydroxylase, beta polypeptide
**Ctsd**	cathepsin D
**ND1**	NADH dehydrogenase subunit 1
**Uba52**	ubiquitin A-52 residue ribosomal protein fusion product 1
**Mmp12**	matrix metallopeptidase 12
**Npc2**	Niemann-Pick disease, type C2
**Actn1**	actinin, alpha 1
**Grem1**	gremlin 1, cysteine knot superfamily, homolog (Xenopus laevis)
**Pgk1**	phosphoglycerate kinase 1
**Col5a2**	collagen, type V, alpha 2
**Grn**	granulin
**Serpine1**	serine (or cysteine) peptidase inhibitor, clade E, member 1
**Arpc2**	actin related protein 2/3 complex, subunit 2
**Lox**	lysyl oxidase
**Eef1b2**	eukaryotic translation elongation factor 1 beta 2
**Prdx1**	peroxiredoxin 1
**Rbm3**	RNA binding motif (RNP1, RRM) protein 3
**Eif4a1**	eukaryotic translation initiation factor 4A1
**Igfbp7**	insulin-like growth factor binding protein 7
**Cd14**	CD14 molecule
**Tpi1**	triosephosphate isomerase 1
**Ptma**	prothymosin alpha
**Sdcbp**	syndecan binding protein
**Pfn1**	profilin 1
**Rab1**	RAB1, member RAS oncogene family
**Eef1g**	eukaryotic translation elongation factor 1 gamma

**Anxa1**	annexin A1
**Atp6v0c**	ATPase, H+ transporting, lysosomal 16kDa, V0 subunit c
**Ubb**	ubiquitin B
**Cyp1b1**	cytochrome P450, family 1, subfamily b, polypeptide 1
**Dynll1**	dynein light chain LC8-type 1

Supplementary Materials and Methods

Sample Preparation for Microarray

Rat HSCs were extracted and culture activated for 7 days. *Sox9* knockdown was achieved using siRNA against two different regions siRNA(A) (cccaccagcgtcagtgaggaa) and siRNA(B) (gagagagactttaagacatta) compared to scrambled control (all from SIGMA). Transfection was achieved using the AMAXA electroporation system as described previously and cells were harvested 48 hours following transfection (2, 4, 5). All samples were verified for extent of knockdown prior to microarray and only samples with >75% *Sox9* abrogation were processed. As a result RNA was isolated from three scrambled and three Sox9 siRNA samples using the RNeasy kit (Qiagen).

Microarray analysis.

Microarray analysis was similar to our previous work (4). RNA quality was assessed by Agilent Bioanalyser. Labelled RNA was hybridized to the Affymetrix Rat Genome 230 2.0 Array platform according to Affymetrix protocols. Background correction, quantile normalization and quantification of gene expression were performed using RMA and differential expression was done with limma in Bioconductor (PMID: 15461798). Differential gene expression was filtered by p value < 0.05 and fold change > +/-1.2 in the 'Sox9-siRNA' versus scrambled control activated HSC groups. Genes were analysed using Database for Annotation, Visualisation and Integrated Discovery (DAVID) version 6.7 and Ingenuity Pathway Analysis (Ingenuity® Systems, www.ingenuity.com). Functional annotation clustering was carried out with medium stringency; functional clusters with an enrichment score > 2 were selected, and proportionately represented by enrichment scores.

Supplementary References

1. Mertin S, McDowall SG, Harley VR. The DNA-binding specificity of SOX9 and other SOX proteins. Nucleic Acids Res 1999;27:1359-1364.

2. Pritchett J, Harvey E, Athwal V, Berry A, Rowe C, Oakley F, Moles A, Mann DA, Bobola N, Sharrocks AD, Thomson BJ, Zaitoun AM, Irving WL, Guha IN, Hanley NA, Hanley KP. Osteopontin is a novel downstream target of SOX9 with diagnostic implications for progression of liver fibrosis in humans. Hepatology 2012;56:1108-1116.

3. Athwal VS, Pritchett J, Llewellyn J, Martin K, Camacho E, Raza SM, Phythian-Adams A, Birchall LJ, Mullan AF, Su K, Pearmain L, Dolman G, Zaitoun AM, Friedman SL, MacDonald A, Irving WL, Guha IN, Hanley NA, Piper Hanley K. SOX9 predicts progression toward cirrhosis in patients while its loss protects against liver fibrosis. EMBO Mol Med 2017;9:1696-1710.

4. Martin K, Pritchett J, Llewellyn J, Mullan AF, Athwal VS, Dobie R, Harvey E, Zeef L, Farrow S, Streuli C, Henderson NC, Friedman SL, Hanley NA, Piper Hanley K. PAK proteins and YAP-1 signalling downstream of integrin beta-1 in myofibroblasts promote liver fibrosis. Nat Commun 2016;7:12502.

5. Piper Hanley K, Oakley F, Sugden S, Wilson DI, Mann DA, Hanley NA. Ectopic SOX9 Mediates Extracellular Matrix Deposition Characteristic of Organ Fibrosis. Journal of Biological Chemistry 2008;283:14063-14071.
